# Association between medical male circumcision and HIV risk compensation among heterosexual men: a systematic review and meta-analysis

**DOI:** 10.1016/S2214-109X(21)00102-9

**Published:** 2021-04-30

**Authors:** Yanxiao Gao, Tanwei Yuan, Yuewei Zhan, Han-Zhu Qian, Yinghui Sun, Weiran Zheng, Leiwen Fu, Bowen Liang, Zhiqiang Zhu, Lin Ouyang, Min Liu, Thomas Fitzpatrick, Zunyou Wu, Xiaojun Meng, Jared M Baeten, Jin Zhao, Sten H Vermund, Maohe Yu, Guohui Wu, Bin Su, Huachun Zou

**Affiliations:** School of Public Health (Shenzhen), Sun Yat-sen University, Shenzhen, China; School of Public Health (Shenzhen), Sun Yat-sen University, Shenzhen, China; School of Public Health (Shenzhen), Sun Yat-sen University, Shenzhen, China; Xiangya Nursing School, Central South University, Changsha, China, Yale School of Public Health, Yale University, New Haven, CT, USA; School of Public Health (Shenzhen), Sun Yat-sen University, Shenzhen, China; School of Public Health (Shenzhen), Sun Yat-sen University, Shenzhen, China; School of Public Health (Shenzhen), Sun Yat-sen University, Shenzhen, China; School of Public Health (Shenzhen), Sun Yat-sen University, Shenzhen, China; Department of Urology, Beijing Youan Hospital, Capital Medical University, Beijing, China; Chongqing Municipal Center for Disease Control and Prevention, Chongqing, China; Department of Urology, Tongren Hospital, Shanghai Jiao Tong University School of Medicine, Shanghai, China; School of Medicine, University of Washington, Seattle, WA, USA; Chinese Center for AIDS and STD Control and Prevention, Chinese Center for Disease Control and Prevention, Beijing, China; Wuxi Municipal Center for Disease Control and Prevention, Wuxi, China; Department of Global Health, Department of Medicine and Department of Epidemiology, University of Washington, Seattle, WA, USA; Shenzhen Municipal Center for Disease Control and Prevention, Shenzhen, China; Yale School of Public Health, Yale University, New Haven, CT, USA; Tianjin Municipal Center for Disease Control and Prevention, Tianjin, China; Chongqing Municipal Center for Disease Control and Prevention, Chongqing, China; Beijing Key Laboratory for HIV/AIDS Research, Center for Infectious Diseases, Beijing Youan Hospital, Capital Medical University, Beijing, China; School of Public Health (Shenzhen), Sun Yat-sen University, Shenzhen, China, Kirby Institute, University of New South Wales, Sydney, NSW, Australia, School of Public Health, Shanghai Jiao Tong University, Shanghai, China

## Abstract

**Background:**

Medical male circumcision (MMC) reduces HIV infection among heterosexual men. There are concerns MMC might prompt higher-risk sexual behaviours because of lower self-perceived risk of HIV infection. We reviewed the published literature to examine associations between MMC and both condom use and number of sex partners among heterosexual men.

**Methods:**

In this systematic review and meta-analysis, we searched PubMed, Embase, and the Cochrane Library for studies published before Nov 15, 2020. Interventional and observational studies were included if they contained original quantitative data describing the association between MMC and condom use or number of sex partners among heterosexual men. We excluded data from men whose circumcisions were ritual or religious and data from men who have sex with men. We extracted odds ratios (ORs) and 95% CIs for the associations between MMC and condomless sex and MMC and multiple sex partners directly from the publications if available, selecting adjusted ORs when provided; when necessary, we calculated ORs and 95% CIs using original study data provided in the publication. We used the Mantel-Haenszel random effects model to calculate pooled ORs and 95% CIs.

**Findings:**

Our search yielded 3035 results, of which 471 were duplicates and 2537 did not meet the inclusion criteria. From the remaining 27 eligible studies, we identified 99 292 men from 31 independent population samples. 24 studies were done in Africa. We found no statistically significant associations between MMC and condomless sex (OR 0·91, 95% CI 0·80–1·05; k=30; *I*^2^=88·7%) or multiple sex partners (1·02, 0·88–1·18; k=27; *I*^2^=90·1%). No associations between MMC and condomless sex or multiple sexual partners were found in any subgroup analyses by study design, income of country, age, recruitment setting, circumcision assessment, circumcision prevalence, and risk of publication bias.

**Interpretation:**

The promotion of circumcision as an HIV preventive measure does not appear to increase higher-risk sexual behaviours in heterosexual men. Ongoing sexual health education should be maintained as a vital component of effective MMC programmes.

## Introduction

Three large randomised controlled trials (RCTs) with a combined total of approximately 11 000 participants, conducted in Africa, showed that medical male circumcision (MMC) could reduce the risk of female-to-male HIV transmission by more than 50%.^1–3^ Immunohistological and histopathological studies have found that there is a high density of HIV target cells in the inner mucosa of the foreskin,^4,5^ suggesting, from a biological perspective, that circumcision could reduce the risk of HIV infection in men. A mathematical model estimated that if 28·8 million men in Africa were to undergo MMC to prevent HIV between 2011 and 2025, 3·4 million new HIV infections could be prevented, saving US$16·5 billion in health-care expenses.^6,7^ WHO and UNAIDS recommended MMC as a key HIV prevention strategy for heterosexual men.

As MMC is promoted as an HIV prevention strategy, an emerging concern is sexual risk compensation among heterosexual men, whereby sexual risk-taking is higher after circumcision, which could offset some or all of the protective benefits of circumcision.^8,9^ After undergoing MMC, men could develop a lower perceived risk of HIV infection, which might lead to higher-risk sexual behaviours, including condomless sex and more sex partners. If heterosexual men have more sex partners and less condom use following circumcision, the result could be an increased transmission of HIV and other sexually transmitted infections.^10^

There is disagreement in the published literature describing associations between MMC and risk compensation. Some studies found that, following MMC, men had lower condom use and a higher number of sex partners.^11–13^ Similarly, risk compensation has been reported in several qualitative studies.^14–16^ However, some studies found that, following MMC, men had a higher frequency of condom use and fewer sex partners.^17,18^ A 2018 meta-analysis of five studies reported no association between MMC and condomless sex among heterosexual men.^19^ A definitive association between MMC and risk compensation remains uncertain. We conducted an updated meta-analysis to explore the association between MMC and condom use and MMC and number of sex partners among heterosexual men.

## Methods

### Search strategy and selection criteria

Our systematic review and meta-analysis were performed according to PRISMA^20^ and MOOSE^21^ guidelines. We searched PubMed, Embase, and the Cochrane Library for studies published in the English language before Nov 15, 2020. We used the following search string combination (“male circumcision” OR “uncircumcised” OR “circumcised”) AND (“condom” OR “condomless” OR “unprotected sex” OR “sexual partner” OR “sex partners” OR “multiple partners” OR “group sex” OR “risk behaviour” OR “risk compensation”). We screened references of eligible full-text articles and relevant review articles for additional eligible publications.

Eligible studies included those that used circumcision status as a study variable and reported condom use or number of sex partners. RCTs, cohort, case-control, and cross-sectional studies, were eligible for inclusion. We excluded studies that only reported on behaviours related to traditional circumcision, given the many different types (ritual or religious) of traditional circumcision in the world, the heterogeneity of technique and completeness of the procedure in these settings, and their typical use before sexual debut. We excluded studies reporting data for men who have sex with men since it is uncertain whether circumcision reduces the risk of male-to-male transmission of HIV. We excluded studies that only included participants who were HIV positive. We included multiple publications from an individual study only when different publications reported data on different independent populations. When one study publication included multiple independent data samples, we reported them separately. Two authors (YG and HZ) independently assessed each study for eligibility. Disagreements were resolved by jointly reviewing the paper in question and in consultation with other coauthors as needed.

### Data analysis

The following information was extracted from each publication selected for inclusion: author, publication year, study country, participant recruitment date, study design, length of follow-up or duration of retrospective assessment, recruitment setting, method of ascertaining circumcision status, sample size, age of circumcised participants (mean or median, as reported), the proportion of circumcised men, frequency of condom use (as per authors’ chosen metric), and number of sex partners (in a lifetime, or in a certain period). Study countries were grouped by WHO regions and World Bank income level.^22^

We used the Newcastle-Ottawa Scale^23^ to assess the methodological quality of cohort studies and used an adapted version of the Newcastle-Ottawa Scale for assessing cross-sectional studies.^24^ We assessed risk of bias in RCTs using the method described in the *Cochrane Handbook of Systematic Reviews of Interventions*.^25,26^ From the papers reviewed, we derived a uniform outcome measure to account for the varying approaches of measuring condom use and the number of sex partners. Detailed methods are presented in the appendix (pp 3–9). We used odds ratios (ORs) and their 95% CIs to describe associations between MMC and condomless sex and MMC and multiple sex partners, with an OR of greater than 1·0 indicating higher risk of increased condomless sex or increased multiple sex partners. We extracted ORs and 95% CIs from publications when possible, selecting adjusted ORs when provided. When necessary, we calculated ORs and 95% CIs using original study data provided in the publication. When ORs for different follow-up timepoints were reported, we extracted the last follow-up timepoint to estimate the overall effect size of the intervention. We did a subgroup analysis of cohort studies and RCTs, in which we included and compared multiple follow-up timepoints from studies. In 2007, MMC was recommended as an effective HIV prevention strategy for heterosexual men so we specifically analysed studies conducted after 2007. As included studies differed in key characteristics, we used the Mantel-Haenszel random effects model to calculate pooled effect sizes. Our primary outcomes were pooled association estimates between MMC and condomless sex and MMC and multiple sex partners.

We used the *I*^2^ statistic to assess the level of statistical heterogeneity between the included studies, with *I*^2^ of less than 50% representing low heterogeneity, between 50% and 75% representing moderate heterogeneity, and greater than 75% representing high heterogeneity.^27^ When substantial heterogeneity was detected, univariate meta-regression analyses were used to investigate sources of heterogeneity. We did subgroup analyses by participant and study characteristics to compare pooled association estimates and heterogeneity. Publication bias was assessed through the Begg’s test and the Egger’s test.^28^ We did sensitivity analyses to detect potential outliers by omitting one estimate at a time and recalculating the pooled estimates. We did our data analyses with Stata version 15.1. Details of the data extraction and analyses are provided in the appendix (p 10).

### Role of the funding source

The funders had no role in study design, data collection, data analysis, data interpretation, or writing of the report.

## Results

**Figure 1:**
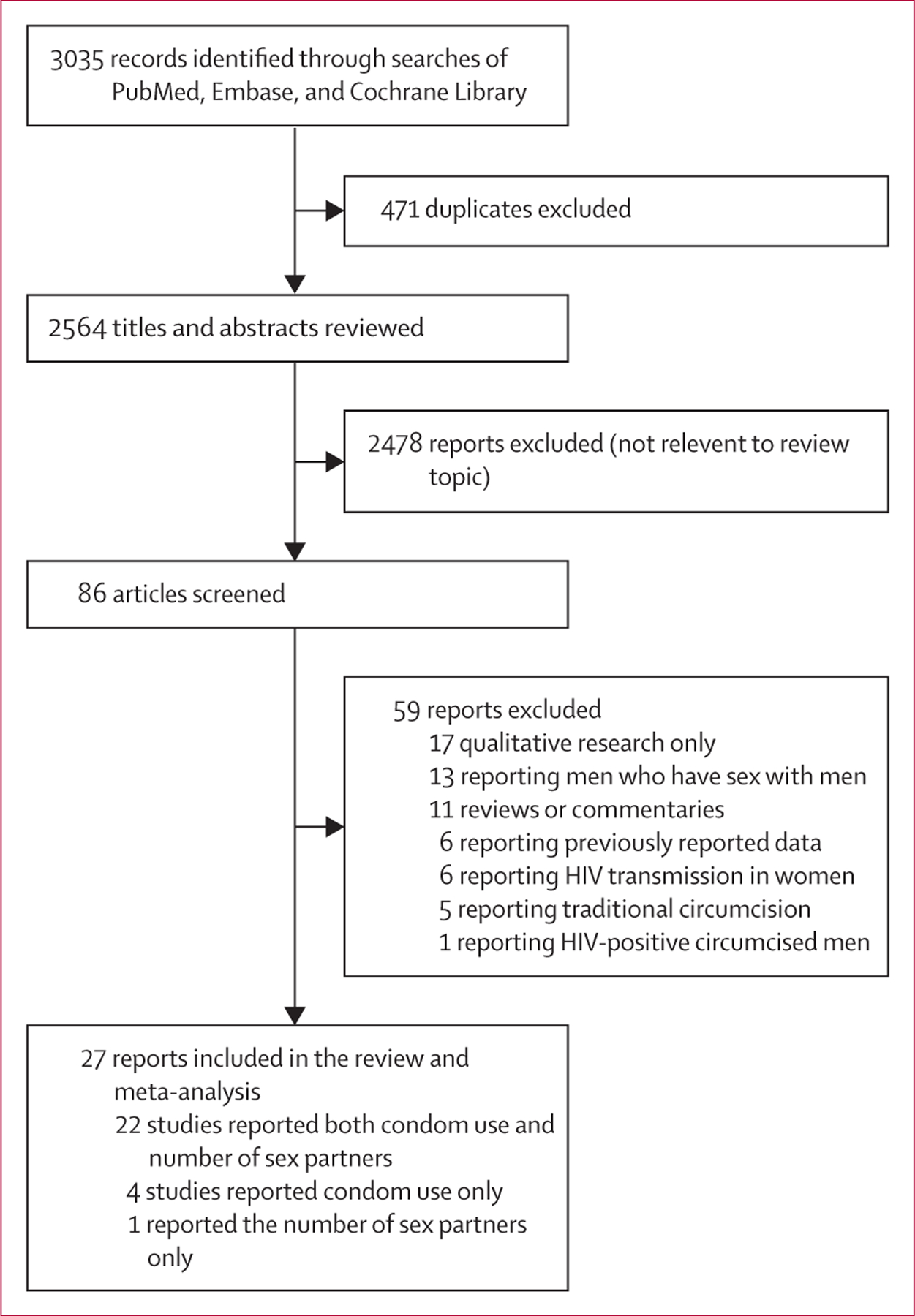
Flowchart of literature search

Our initial search yielded 3035 results, of which 471 were duplicates. Of the remaining 2564 titles and abstracts reviewed, 2478 (82%) did not meet the inclusion criteria. We did a full-text review of 86 (3%) of the 2564 articles, of which 59 were excluded (figure 1). 27 (1%) articles,^2,3,11–13,17,18,29–48^ including 31 samples of 99 292 heterosexual men, were eligible for our analysis. Of these, 24 studies were from Africa,^2,3,11–13,17,18,29–34,36,37,39–44,46–48^ one from Europe,^38^ one from the Western Pacific region,^45^ and one from the Americas.^35^ Four studies^36,39,42,47^ reported condom use only, one study reported number of sex partners only (≥2 *vs* ≤1),^38^ and 22 studies^2,3,11–13,17,18,29–35,37,40,41,43–46,48^ reported both condom use and number of sex partners. 11 (41%) of the 27 studies were cohort studies or RCTs, and 16 (59%) were cross-sectional. Detailed study information is presented in the appendix (pp 11–19).

Included studies were conducted between 1997 and 2018 and published between 1999 and 2020. The number of participants enrolled in each study ranged from 194 to 9983. Mean ages of circumcised participants ranged from 16·8 to 30·8 years (number of estimates [k]=9), and median ages varied from 17·0 to 32·0 years (k=12). The proportion of circumcised men ranged from 5·3% to 85·8% (median 45·0%, IQR 24·1–50·0; k=31). The proportion of men who had condomless sex ranged from 12·0% to 93·0% (median 61·0%, IQR 46·2–75·2; k=30) in cohort, RCT, and cross-sectional studies, and 18·5% to 93·0% (61·3%, 50·2–73·1; k=11) in RCTs or cohort studies. The proportion of men who had had at least two sexual partners ranged from 2·2% to 80·5% (median 28·9%, IQR 13·0–40·5; k=27) in cohort, RCT, and cross-sectional studies, and 2·2% to 59·5% (32·0%, 25·4–36·8; k=10) in RCT or cohort studies.

**Figure 2:**
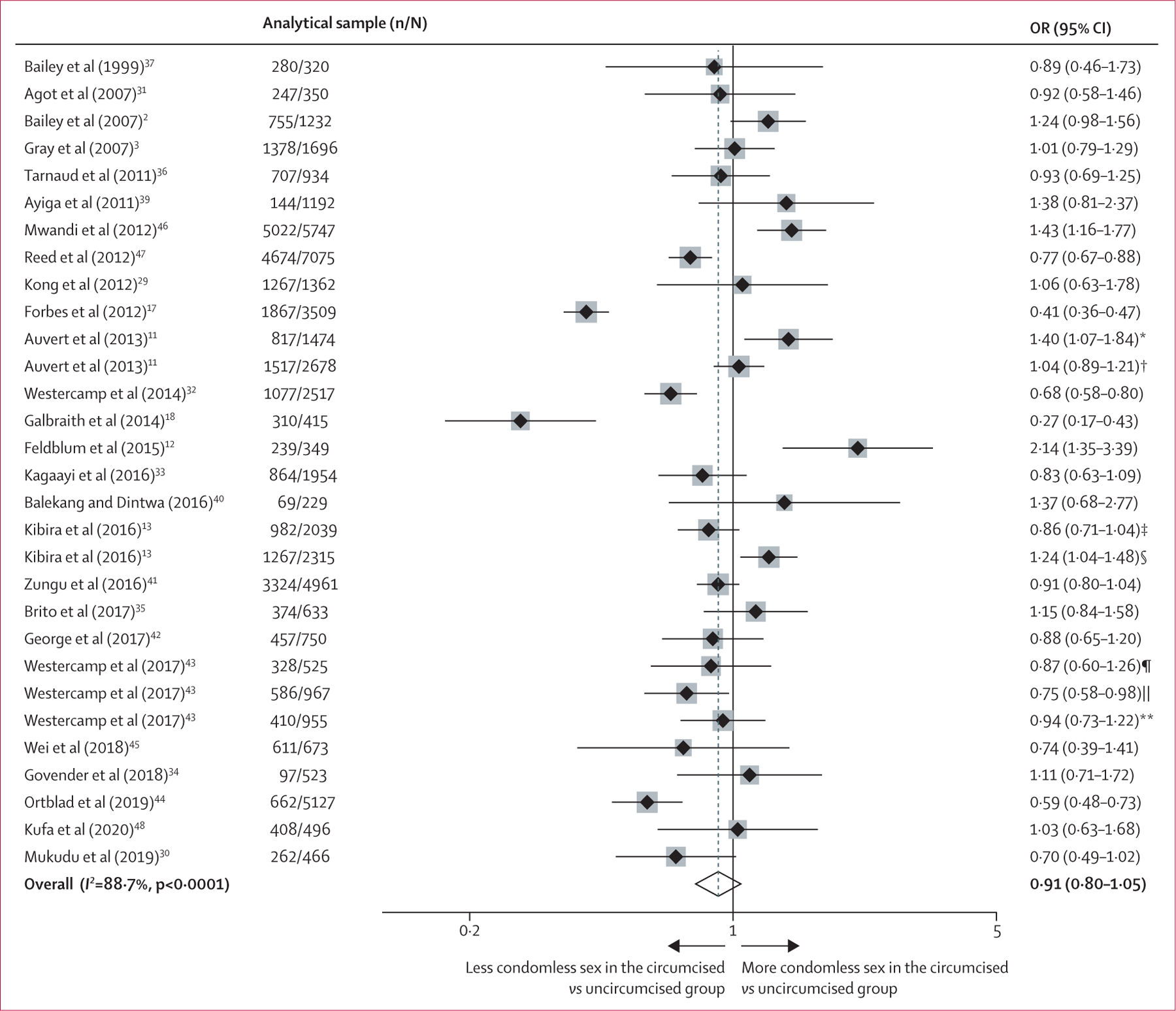
Meta-analysis of the association between medical male circumcision and condomless sex among heterosexual men OR=odds ratio. *Based on data from Auvert and colleagues’ 2007–08 study. †Based on data from Auvert and colleagues’ 2010–11 study. ‡Based on data from Kibira and colleagues’ 2004 study. §Based on data from Kibira and colleagues’ 2011 study. ¶Based on data from Westercamp and colleagues’ 2008–09 study. ||Based on data from Westercamp and colleagues’ 2011 study. **Based on data from Westercamp and colleagues’ 2013 study.

**Figure 3:**
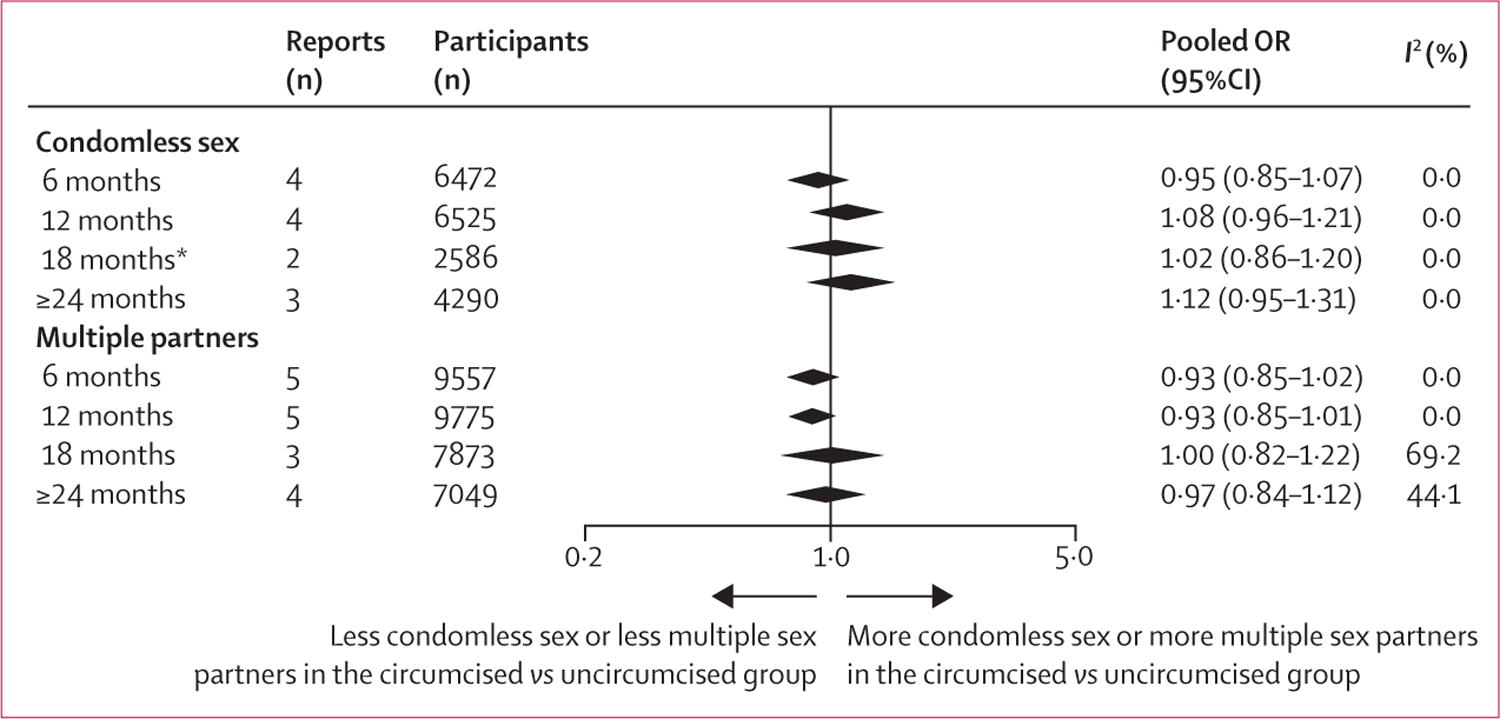
Association between medical male circumcision and condomless sex or multiple sex partners among heterosexual men, stratified by follow-up time Pooled OR less than 1·0 indicates reduction of outcomes in the circumcised group compared with the uncircumcised group. OR=odds ratio. *One of the reports is a follow-up visit at 21 months from a randomised controlled trial.

**Figure 4:**
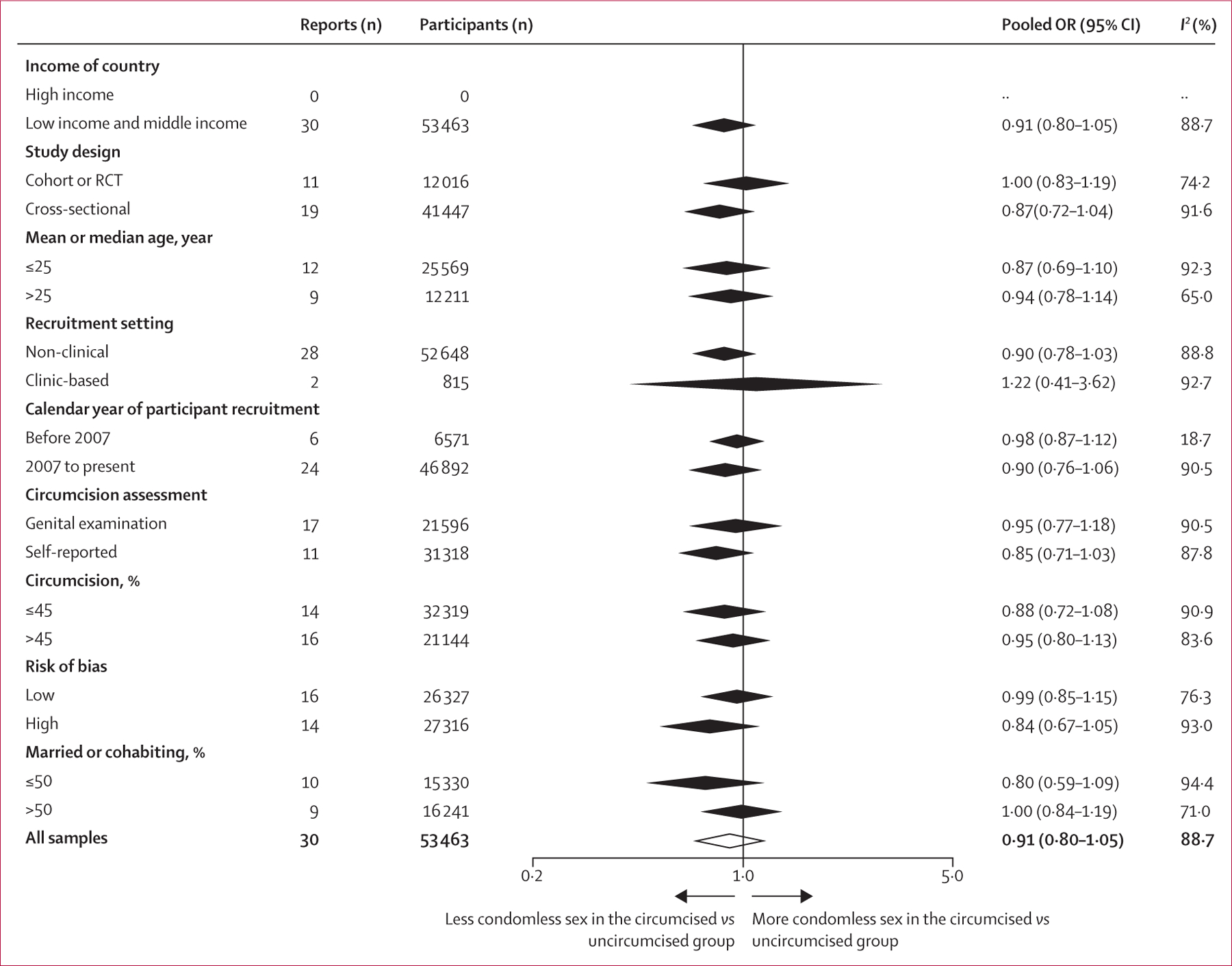
Subgroup meta-analyses of the association between medical male circumcision and condomless sex among heterosexual men OR=odds ratio. RCT=randomised control trial.

26 studies, including 30 samples of 93 897 heterosexual men, assessed the association between MMC and condomless sex. All men included in these studies lived in low-income and middle-income countries (LMICs). MMC was not significantly associated with condomless sex (OR 0·91, 95% CI 0·80–1·05; k=30; *I*^2^=88·7%; figure 2). Subgroup analysis of six cohort and RCT studies^2,3,29,31,34,36^ showed no statistically significant change in condomless sex among circumcised men across different follow-up timepoints (figure 3). Subgroup analyses also showed no statistically significant association between MMC and condomless sex by age, recruitment setting, circumcision assessment, circumcision prevalence, married or cohabiting prevalence, risk of bias, or study design (figure 4). Subgroup analyses of studies conducted after 2007 showed no statistically significant association between MMC and condomless sex (appendix p 24).

**Figure 5:**
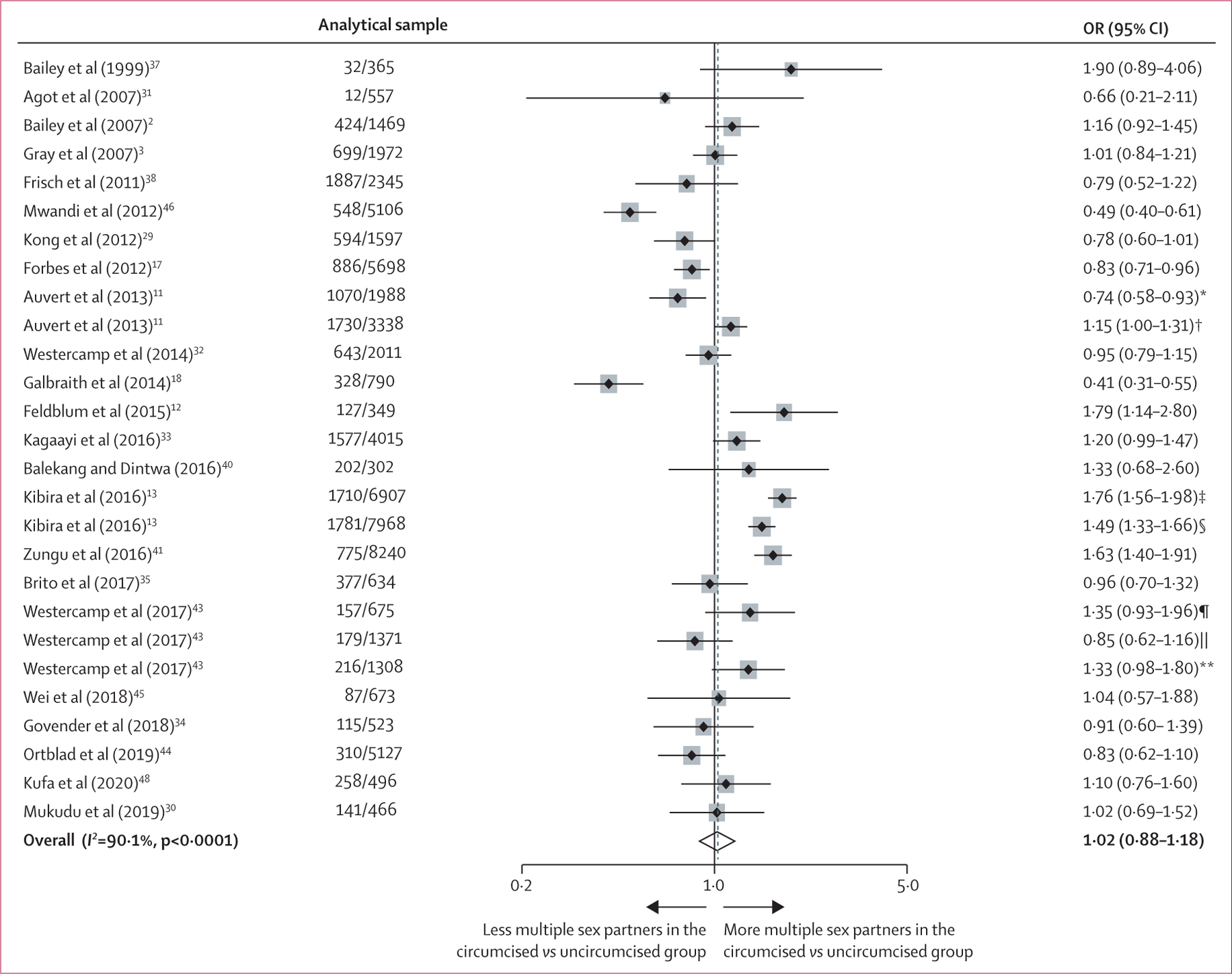
Meta-analysis of the association between medical male circumcision and multiple sex partners among heterosexual men OR=odds ratio. *Based on data from Auvert and colleagues’ 2007–08 study. †Based on data from Auvert and colleagues’ 2010–11 study. ‡Based on data from Kibira and colleagues’ 2004 study. §Based on data from Kibira and colleagues’ 2011 study. ¶Based on data from Westercamp and colleagues’ 2008–09 study. ||Based on data from Westercamp and colleagues’ 2011 study. **Based on data from Westercamp and colleagues’ 2013 study.

**Figure 6:**
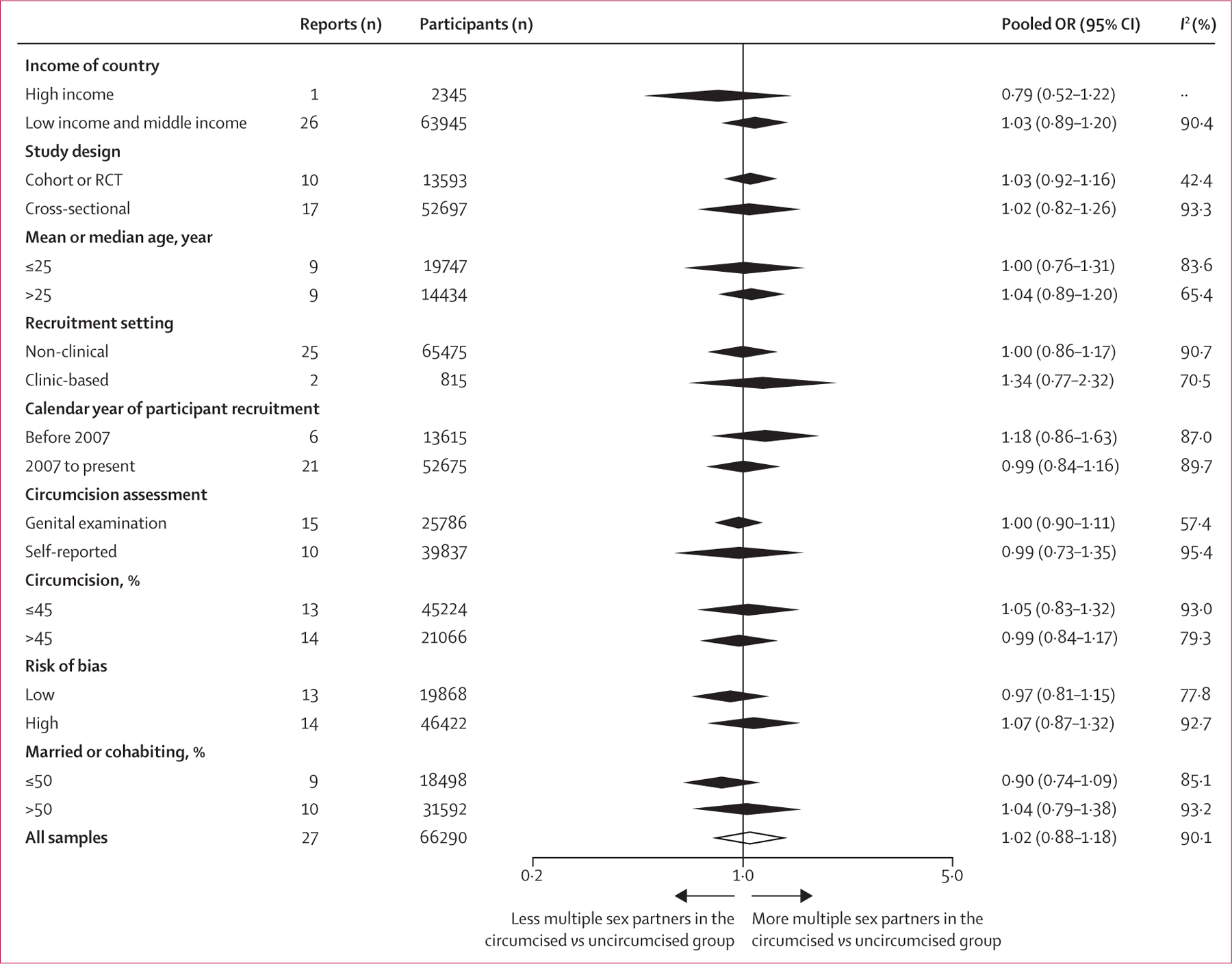
Subgroup meta-analyses of the association between medical male circumcision and multiple sex partners among heterosexual men OR=odds ratio. RCT=randomised controlled trial.

23 studies, including 27 samples of 88 457 participants, reported the association between MMC and number of sex partners. We found no statistically significant association between MMC and multiple sex partners (OR 1·02, 95% CI 0·88–1·18; k=27; *I*^2^=90·1%; figure 5). Subgroup analysis of seven cohort studies and RCTs^2,3,29,31–34^ found no statistically significant change in multiple sex partners among circumcised men across different follow-up time points (figure 3). There was no statistically significant difference between MMC and multiple sexual partners in subgroup analysis by country level of income, study design, age, recruitment setting, circumcision assessment, circumcision prevalence, married or cohabiting prevalence, or risk of bias (figure 6). Subgroup analyses of studies conducted after 2007 showed no statistically significant association between MMC and multiple sexual partners (appendix p 24).

We tested publication bias in studies that reported condomless sex (Begg’s test p=0·80, Egger’s test p=0·19) and multiple sex partners (p=0·74, p=0·079; appendix pp 27–28). Sensitivity analyses did not show any individual study had a significant disproportionate effect on the pooled association estimates between MMC and condomless sex or MMC and multiple sex partners (appendix pp 25–26). Significant heterogeneity was found in studies that reported condomless sex (*I*^2^=88·7%; figure 4) and multiple sex partners (*I*^2^=90·1%; figure 6). The high level of heterogeneity in studies that reported condomless sex was substantially lower, or absent, in study subgroups in which participants were recruited before 2007, and in follow-ups of cohort studies or RCTs at 6 months, 12 months, 18 months, and 24 months or later (*I*^2^ range 0–18·7%; figures 3, 4). For studies that reported multiple sex partners, low heterogeneity was seen in the following subgroups: follow-up of cohort studies or RCTs at 6 months, 12 months, and 24 months or later (*I*^2^ range 0–44·1%; figures 3, 6).

## Discussion

Our systematic review and meta-analysis found that MMC was not associated with increased condomless sex or multiple sex partners among heterosexual men. This lack of association persisted across a wide variety of subgroups. These findings might help alleviate concerns that widespread MMC programmes could lead to risk compensation and, therefore, reduce the benefit of MMC.

Our finding that MMC was not associated with more condomless sex is consistent with a previous meta-analysis of five cohort and experimental studies.^19^ Our review included 22 additional studies, which enabled detailed subgroup analyses. Although our results were statistically insignificant, subgroup analyses of cohort studies and RCTs suggested that, following the MMC procedure, the point estimate of the OR changed slightly from 0·95 at 6 months to 1·12 at 24 months or later. A possible reason behind this finding is that many large circumcision campaigns in Africa included free condom provision and health education to promote condom use. However, the positive effect of the health education component of these interventions on circumcised men might have declined over time. These results suggest sustained sexual health education is necessary after surgery.

We found no overall association between MMC and multiple sex partners. However, subgroup analyses of cohort studies and RCTs suggested that, following the MMC procedure, the point estimate of the OR changed slightly from 0·93 at 12 months or earlier to 1·00 at 18 months and 0·97 at 24 months or later. This slight change could be because circumcised men were advised to abstain from sex in the first few months after surgery or received a package of complementary risk-reduction services following the procedure.^49^ Circumcised men had a shorter follow-up time (about 6 weeks less) than uncircumcised men.^1–3^ The effect of education on sexual risk behaviours among circumcised men might have declined over time, explaining why there was no change in multiple sex partners between circumcised and uncircumcised men after 12 months.

The possibility that male circumcision might reduce risk of HIV acquisition was first proposed in 1986.^50,51^ Before 2007, the effectiveness of MMC in reducing HIV infection was not well understood among either health-care workers, policy makers, or the general population. The three large-scale African RCTs that ended in late 2006^1–3^ confirmed the high degree of efficacy of MMC in reducing HIV acquisition in heterosexual men. In 2007, WHO and UNAIDS recommended that MMC be offered as part of a comprehensive HIV prevention strategy in areas with high prevalence of heterosexually transmitted HIV and low rates of male circumcision. We found no association between MMC and condomless sex or MMC and multiple sex partners, either before or after 2007. Similarly, subgroup analyses of studies after 2007 showed no association between MMC and either type of risk compensation. Based on the WHO and UNAIDS recommendation, expanding access to safe MMC services is considered a priority in 14 countries in eastern Africa and southern Africa. MMC services are provided as a package of prevention measures, including safer sex education, condom education and provision, and HIV testing. We found no association between MMC and risk compensation in these priority countries (appendix p 24), and the odds of condomless sex (from 0·98 to 0·90) and multiple sex partners (from 1·18 to 0·99) slightly decreased in circumcised men in these priority countries compared with that before 2007 (appendix p 24; figures 4, 6). These findings could be because circumcised men accepted and benefited from a package of male circumcision services. It is plausible that the perceived protective effects of MMC do not significantly change sexual risk behaviours among circumcised men.

Modelling studies suggest that circumcising 80% of HIV-negative men aged 15–49 years within 15 years could prevent 3·4 million incident infections in LMICs.^6,7,52^ Difficulties exist in scaling up MMC programmes, including shortage of staff and capacity, limitations on clinical space and available equipment in some LMICs,^53,54^ and difficulties in implementing circumcision techniques as recommended by WHO and UNAIDS.^55^ From the standpoint of individuals, there remain several barriers to the uptake of MMC, including fear of pain, concerns about sexual abstinence after surgery, resistance from female partners, post-surgical sexual performance and satisfaction, cultural factors, and the loss of wages during and after the procedure.^53,56–58^ Another major barrier to MMC programmes is concern about risk compensation after surgery. However, a 2020 systematic review reported that circumcised men did not have higher-risk sex than uncircumcised men.^59^ Our results showed no evidence of post-MMC disinhibition in LMICs. The odds of having multiple sex partners among circumcised men was not notably different from that among uncircumcised men in LMICs. We found that there were no significant differences in condomless sex between circumcised men and uncircumcised men in LMICs, which could be due to globally supported HIV prevention campaigns in these countries.^60^

Findings from this meta-analysis are largely consistent with previous qualitative research on the same topic, with most men reporting that they adopt protective sexual behaviours (increase condom use or reduce the number of sexual partners) or maintained protective sexual behaviours after circumcision.^61–65^ However, some studies reported that circumcised men increased higher-risk sexual behaviours after circumcision because they falsely believed that MMC could offer complete protection from HIV infection,^16,66,67^ and one study from Zambia reported that about 30–40% of women incorrectly believed MMC fully protects men from getting HIV.^68^ In addition, some studies found that men were more likely to report higher-risk sexual behaviours after MMC or report false belief regarding the protective effect provided by circumcision if they had low levels of education, were married, had alcoholism, or had misconceptions regarding antiretroviral therapy; religious beliefs might also affect risk behaviour after MMC.^39,69^ Therefore, misconceptions about the effect of MMC indicate the need to strengthen risk reduction education, especially for specific groups, including those with lower education, married men, and men with alcoholism.

Our study has several limitations. First, about two-fifths of the included studies could be subject to high risk of bias, with high heterogeneity across these studies. Consequently, our results should be interpreted with caution. However, higher-quality studies did not provide substantial evidence of risk compensation for MMC in subgroup analyses. The Oxford Centre for Evidence-Based Medicine clearly states that not all systematic reviews with statistically significant heterogeneity need be worrisome, and not all worrisome heterogeneity need be statistically significant.^70^ The public health implication derived from the results is more important than the statistical heterogeneity. Previous modelling studies reported that the benefit of MMC would only be offset when the rate of having multiple sexual partners increased by more than 200%,^71^ or that of having condomless sex rises by 50%.^72^ In this review, only one study reported a decrease in the rate of condom use by more than 50%,^12^ and none reported an increase in the rate of having multiple partners by more than 200%, supporting the reliability of our findings. Second, since studies defined condomless sex in many ways, we redefined the primary outcome variables of reported condom use status during any sexual activity from a three-category variable (ie, consistent condom use *vs* inconsistent condom use *vs* no condom use) to a two-category variable (ie, inconsistent or no condom use *vs* consistent condom use) in the analyses, which might contribute to heterogeneity among studies. Nevertheless, a previous meta-analysis defined inconsistent condom use differently from our definition,^19^ and also found no evidence of risk compensation. Third, data on condom use and number of sex partners were self-reported. Fourth, there is some evidence of publication bias according to Egger’s test and the funnel plot, which means statistically significant results are more likely to be published than non-significant results. As a result, recall bias and social desirability bias could be involved.^73^

In conclusion, our meta-analysis supports efforts to scale up MMC as an HIV prevention measure. This intervention is unlikely to encourage higher-risk sexual behaviours among heterosexual men. Nonetheless, to ensure that risk compensation is minimised, global programmes must strengthen long-term health education interventions within circumcision programmes.

## Supplementary Material

1

## Research in context

### Evidence before this study

We searched PubMed, Embase, and the Cochrane Library using the terms (“male circumcision” OR “uncircumcised” OR “circumcised”) AND (“condom” OR “condomless” OR “unprotected sex” OR “sexual partner” OR “sex partners” OR “multiple partners” OR “group sex” OR “risk behavior” OR “risk compensation”), for original studies, as well as systematic reviews and meta-analyses, published in the English language from inception to Nov 15, 2020. We found one previous meta-analysis, published in 2018, that found that medical male circumcision (MMC) was not associated with a change in condom use in the short term or medium term. This previous study included 16 521 heterosexual men from five studies. Association between MMC and multiple sex partners was not reported.

### Added value of this study

Our systematic review of the literature reporting the association between MMC and risk compensation among men (excluding men who have sex with men) included 99 292 heterosexual men from 27 studies, 22 of which had not been included in the existing meta-analysis. Our results showed no association between MMC and condomless sex or MMC and multiple sex partners among heterosexual men. Statistically significant associations were not found in subgroup analyses.

### Implications of all the available evidence

Our systematic review and meta-analysis found that MMC is unlikely to encourage higher risk sexual behaviours among heterosexual men. This finding is encouraging to the scale-up of MMC as a proven HIV prevention measure. Nonetheless, it is necessary to maintain sexual health education after MMC.

## References

[1] Auvert B, Taljaard D, Lagarde E, Sobngwi-Tambekou J, Sitta R, Puren A. Randomized, controlled intervention trial of male circumcision for reduction of HIV infection risk: the ANRS 1265 trial. PLoS Med 2005; 2: e298. 16231970 10.1371/journal.pmed.0020298 PMC1262556

[2] Bailey RC, Moses S, Parker CB, Male circumcision for HIV prevention in young men in Kisumu, Kenya: a randomised controlled trial. Lancet 2007; 369: 643–56. 17321310 10.1016/S0140-6736(07)60312-2

[3] Gray RH, Kigozi G, Serwadda D, Male circumcision for HIV prevention in men in Rakai, Uganda: a randomised trial. Lancet 2007; 369: 657–66. 17321311 10.1016/S0140-6736(07)60313-4

[4] McCoombe SG, Short RV. Potential HIV-1 target cells in the human penis. AIDS 2006; 20: 1491–95. 16847403 10.1097/01.aids.0000237364.11123.98

[5] Patterson BK, Landay A, Siegel JN, Susceptibility to human immunodeficiency virus-1 infection of human foreskin and cervical tissue grown in explant culture. Am J Pathol 2002; 161: 867–73. 12213715 10.1016/S0002-9440(10)64247-2 PMC1867269

[6] Njeuhmeli E, Forsythe S, Reed J, Voluntary medical male circumcision: modeling the impact and cost of expanding male circumcision for HIV prevention in eastern and southern Africa. PLoS Med 2011; 8: e1001132. 22140367 10.1371/journal.pmed.1001132 PMC3226464

[7] Hankins C, Forsythe S, Njeuhmeli E. Voluntary medical male circumcision: an introduction to the cost, impact, and challenges of accelerated scaling up. PLoS Med 2011; 8: e1001127. 22140362 10.1371/journal.pmed.1001127 PMC3226452

[8] Dushoff J, Patocs A, Shi CF. Modeling the population-level effects of male circumcision as an HIV-preventive measure: a gendered perspective. PLoS One 2011; 6: e28608. 22205956 10.1371/journal.pone.0028608 PMC3243682

[9] Rojas Castro D, Delabre RM, Molina JM. Give PrEP a chance: moving on from the “risk compensation” concept. J Int AIDS Soc 2019; 22 (suppl 6): e25351. 31468693 10.1002/jia2.25351 PMC6715948

[10] Cassell MM, Halperin DT, Shelton JD, Stanton D. Risk compensation: the Achilles’ heel of innovations in HIV prevention? BMJ 2006; 332: 605–07. 16528088 10.1136/bmj.332.7541.605 PMC1397752

[11] Auvert B, Taljaard D, Rech D, Association of the ANRS-12126 male circumcision project with HIV levels among men in a South African township: evaluation of effectiveness using cross-sectional surveys. PLoS Med 2013; 10: e1001509. 24019763 10.1371/journal.pmed.1001509 PMC3760784

[12] Feldblum PJ, Okech J, Ochieng R, Longer-term follow-up of Kenyan men circumcised using the ShangRing device. PLoS One 2015; 10: e0137510. 26367141 10.1371/journal.pone.0137510 PMC4569077

[13] Kibira SP, Sandøy IF, Daniel M, Atuyambe LM, Makumbi FE. A comparison of sexual risk behaviours and HIV seroprevalence among circumcised and uncircumcised men before and after implementation of the safe male circumcision programme in Uganda. BMC Public Health 2016; 16: 7. 26727935 10.1186/s12889-015-2668-3 PMC4700673

[14] Mantell JE, Smit JA, Saffitz JL, Medical male circumcision and HIV risk: perceptions of women in a higher learning institution in KwaZulu-Natal, South Africa. Sex Health 2013; 10: 112–18. 23448912 10.1071/SH12067 PMC3963517

[15] Ombere SO, Nyambedha EO, Bukachi SA. Wimbo: implications for risk of HIV infection among circumcised fishermen in western Kenya. Cult Health Sex 2015; 17: 1147–54. 25774858 10.1080/13691058.2015.1018949

[16] Humphries H, van Rooyen H, Knight L, Barnabas R, Celum C. ‘If you are circumcised, you are the best’: understandings and perceptions of voluntary medical male circumcision among men from KwaZulu-Natal, South Africa. Cult Health Sex 2015; 17: 920–31. 25567140 10.1080/13691058.2014.992045 PMC4470729

[17] Forbes HJ, Doyle AM, Maganja K, Rapid increase in prevalence of male circumcision in rural Tanzania in the absence of a promotional campaign. PLoS One 2012; 7: e40507. 22792359 10.1371/journal.pone.0040507 PMC3391251

[18] Galbraith JS, Ochieng A, Mwalili S, Status of voluntary medical male circumcision in Kenya: findings from 2 nationally representative surveys in Kenya, 2007 and 2012. J Acquir Immune Defic Syndr 2014; 66 (suppl 1): S37–45. 24732820 10.1097/QAI.0000000000000121 PMC4794989

[19] Kabwama SN, Ssewanyana D, Berg-Beckhoff G. The association between male circumcision and condom use behavior—a meta-analysis. Mater Sociomed 2018; 30: 62–66. 29670480 10.5455/msm.2018.30.62-66 PMC5857052

[20] Liberati A, Altman DG, Tetzlaff J, The PRISMA statement for reporting systematic reviews and meta-analyses of studies that evaluate healthcare interventions: explanation and elaboration. BMJ 2009; 339: b2700. 19622552 10.1136/bmj.b2700 PMC2714672

[21] Stroup DF, Berlin JA, Morton SC, Meta-analysis of observational studies in epidemiology: a proposal for reporting. Meta-analysis Of Observational Studies in Epidemiology (MOOSE) group. JAMA 2000; 283: 2008–12. 10789670 10.1001/jama.283.15.2008

[22] World Bank Data Team. New country classifications by income level: 2019–2020. July 1, 2019. https://blogs.worldbank.org/opendata/new-country-classifications-income-level-2019–2020 (accessed Nov 18, 2020).

[23] Wells GA, Shea B, O’Connell D, The Newcastle-Ottawa Scale (NOS) for assessing the quality of nonrandomised studies in meta-analyses. 2019. http://www.ohri.ca/programs/clinical_epidemiology/oxford.asp (accessed Nov 16, 2020).

[24] Herzog R, Álvarez-Pasquin MJ, Díaz C, Del Barrio JL, Estrada JM, Gil Á. Are healthcare workers’ intentions to vaccinate related to their knowledge, beliefs and attitudes? A systematic review. BMC Public Health 2013; 13: 154. 23421987 10.1186/1471-2458-13-154 PMC3602084

[25] Siegfried N, Muller M, Deeks JJ, Volmink J. Male circumcision for prevention of heterosexual acquisition of HIV in men. Cochrane Database Syst Rev 2009; 2: CD003362. 10.1002/14651858.CD003362.pub2 PMC11666075 19370585

[26] Higgins JTJ, Thomas J, Chandler J, Cochrane handbook for systematic reviews of interventions. 2019. https://training.cochrane.org/handbook/current (accessed Nov 16 2020).

[27] Higgins JP, Thompson SG, Deeks JJ, Altman DG. Measuring inconsistency in meta-analyses. BMJ 2003; 327: 557–60. 12958120 10.1136/bmj.327.7414.557 PMC192859

[28] Egger M, Davey Smith G, Schneider M, Minder C. Bias in meta-analysis detected by a simple, graphical test. BMJ 1997; 315: 629–34. 9310563 10.1136/bmj.315.7109.629 PMC2127453

[29] Kong X, Kigozi G, Nalugoda F, Assessment of changes in risk behaviors during 3 years of posttrial follow-up of male circumcision trial participants uncircumcised at trial closure in Rakai, Uganda. Am J Epidemiol 2012; 176: 875–85. 23097257 10.1093/aje/kws179 PMC3626062

[30] Mukudu H, Dietrich J, Otwombe K, Voluntary medical male circumcision (VMMC) for prevention of heterosexual transmission of HIV and risk compensation in adult males in Soweto: findings from a programmatic setting. PLoS One 2019; 14: e0213571. 30845185 10.1371/journal.pone.0213571 PMC6405100

[31] Agot KE, Kiarie JN, Nguyen HQ, Odhiambo JO, Onyango TM, Weiss NS. Male circumcision in Siaya and Bondo Districts, Kenya: prospective cohort study to assess behavioral disinhibition following circumcision. J Acquir Immune Defic Syndr 2007; 44: 66–70. 17019365 10.1097/01.qai.0000242455.05274.20

[32] Westercamp N, Agot K, Jaoko W, Bailey RC. Risk compensation following male circumcision: results from a two-year prospective cohort study of recently circumcised and uncircumcised men in Nyanza Province, Kenya. AIDS Behav 2014; 18: 1764–75. 25047688 10.1007/s10461-014-0846-4

[33] Kagaayi J, Kong X, Kigozi G, Self-selection of male circumcision clients and behaviors following circumcision in a service program in Uganda. AIDS 2016; 30: 2125–29. 27203716 10.1097/QAD.0000000000001169 PMC5035768

[34] Govender K, George G, Beckett S, Montague C, Frohlich J. Risk compensation following medical male circumcision: results from a 1-year prospective cohort study of young school-going men in KwaZulu-Natal, South Africa. Int J Behav Med 2018; 25: 123–30. 28688094 10.1007/s12529-017-9673-0

[35] Brito MO, Khosla S, Pananookooln S, Sexual pleasure and function, coital trauma, and sex behaviors after voluntary medical male circumcision among men in the Dominican Republic. J Sex Med 2017; 14: 526–34. 28258953 10.1016/j.jsxm.2017.01.020

[36] Tarnaud C, Lissouba P, Cutler E, Puren A, Taljaard D, Auvert B. Association of low-risk human papillomavirus infection with male circumcision in young men: results from a longitudinal study conducted in Orange Farm (South Africa). Infect Dis Obstet Gynecol 2011; 2011: 567408. 21584275 10.1155/2011/567408 PMC3092496

[37] Bailey RC, Neema S, Othieno R. Sexual behaviors and other HIV risk factors in circumcised and uncircumcised men in Uganda. J Acquir Immune Defic Syndr 1999; 22: 294–301. 10770351 10.1097/00126334-199911010-00012

[38] Frisch M, Lindholm M, Grønbæk M. Male circumcision and sexual function in men and women: a survey-based, cross-sectional study in Denmark. Int J Epidemiol 2011; 40: 1367–81. 21672947 10.1093/ije/dyr104

[39] Ayiga N, Letamo G. Impact of male circumcision on HIV risk compensation through the impediment of condom use in Botswana. Afr Health Sci 2011; 11: 550–59. 22649434 PMC3362967

[40] Balekang GB, Dintwa KF. A comparison of risky sexual behaviours between circumcised and uncircumcised men aged 30–44 years in Botswana. Afr Health Sci 2016; 16: 105–15. 27358620 10.4314/ahs.v16i1.14 PMC4915434

[41] Zungu NP, Simbayi LC, Mabaso M, HIV risk perception and behavior among medically and traditionally circumcised males in South Africa. BMC Public Health 2016; 16: 357. 27112917 10.1186/s12889-016-3024-y PMC4845373

[42] George G, Govender K, Beckett S, Montague C, Frohlich J. Factors associated with the take-up of voluntary medical male circumcision amongst learners in rural KwaZulu-Natal. Afr J AIDS Res 2017; 16: 251–56. 28978292 10.2989/16085906.2017.1369441

[43] Westercamp M, Jaoko W, Mehta S, Abuor P, Siambe P, Bailey RC. Changes in male circumcision prevalence and risk compensation in the Kisumu, Kenya population, 2008–2013. J Acquir Immune Defic Syndr 2017; 74: e30–37. 27632232 10.1097/QAI.0000000000001180 PMC5233580

[44] Ortblad KF, Harling G, Chimbindi N, Tanser F, Salomon JA, Bärnighausen T. Does incident circumcision lead to risk compensation? Evidence from a population cohort in KwaZulu-Natal, South Africa. J Acquir Immune Defic Syndr 2019; 80: 269–75. 30531298 10.1097/QAI.0000000000001912 PMC6375765

[45] Wei FX, Guo M, Ma XJ, The impact of male circumcision on the natural history of genital HPV infection: a prospective cohort study. Zhonghua Yu Fang Yi Xue Za Zhi 2018; 52: 486–92 (in Chinese). 29747340 10.3760/cma.j.issn.0253-9024.2018.05.007

[46] Mwandi Z, Bunnell R, Cherutich P, Male circumcision programmes in Kenya: lessons from the Kenya AIDS Indicator Survey 2007. Bull World Health Organ 2012; 90: 642–51. 22984308 10.2471/BLT.11.096412 PMC3442396

[47] Reed JB, Mirira M, Grund J, Male circumcision in Swaziland: demographics, behaviours and HIV prevalence. J Int AIDS Soc 2012; 15: 137.

[48] Kufa T, Radebe F, Maseko V, Puren A, Kularatne R. Medical male circumcision and associations among sexually transmitted infections service attendees. AIDS Behav 2020; 24: 1422–31. 31720907 10.1007/s10461-019-02729-9

[49] Dickson KE, Reed J, Rech D. Considerations for implementing models for optimizing the volume and efficiency of male circumcision services. Field testing edn. Geneva, Switzerland: World Health Organization, 2010.

[50] Fink AJ. A possible explanation for heterosexual male infection with AIDS. N Engl J Med 1986; 315: 1167. 3762636

[51] Alcena V. AIDS in Third World countries. N Y State J Med 1986; 86: 446. 3463895

[52] Sgaier SK, Reed JB, Thomas A, Njeuhmeli E. Achieving the HIV prevention impact of voluntary medical male circumcision: lessons and challenges for managing programs. PLoS Med 2014; 11: e1001641. 24800840 10.1371/journal.pmed.1001641 PMC4011573

[53] Herman-Roloff A, Llewellyn E, Obiero W, Implementing voluntary medical male circumcision for HIV prevention in Nyanza Province, Kenya: lessons learned during the first year. PLoS One 2011; 6: e18299. 21483697 10.1371/journal.pone.0018299 PMC3070734

[54] Tynan A, Vallely A, Kelly A, Health workers, health facilities and penile cutting in Papua New Guinea: implications for male circumcision as an HIV prevention strategy. P N G Med J 2011; 54: 109–22. 24494507

[55] Cheng F, Lü NQ, Xu HQ, Clinical studies of shang ring male circumcision in China and Africa. Zhonghua Nan Ke Xue 2014; 20: 291–98 (in Chinese). 24873152

[56] Westercamp N, Bailey RC. Acceptability of male circumcision for prevention of HIV/AIDS in sub-Saharan Africa: a review. AIDS Behav 2007; 11: 341–55. 17053855 10.1007/s10461-006-9169-4 PMC1847541

[57] Herman-Roloff A, Otieno N, Agot K, Ndinya-Achola J, Bailey RC. Acceptability of medical male circumcision among uncircumcised men in Kenya one year after the launch of the national male circumcision program. PLoS One 2011; 6: e19814. 21603622 10.1371/journal.pone.0019814 PMC3095626

[58] Ssekubugu R, Leontsini E, Wawer MJ, Contextual barriers and motivators to adult male medical circumcision in Rakai, Uganda. Qual Health Res 2013; 23: 795–804. 23515302 10.1177/1049732313482189

[59] Atkins K, Yeh PT, Kennedy CE, Service delivery interventions to increase uptake of voluntary medical male circumcision for HIV prevention: a systematic review. PLoS One 2020; 15: e0227755. 31929587 10.1371/journal.pone.0227755 PMC6957297

[60] Sullivan PS, Carballo-Diéguez A, Coates T, Successes and challenges of HIV prevention in men who have sex with men. Lancet 2012; 380: 388–99. 22819659 10.1016/S0140-6736(12)60955-6 PMC3670988

[61] L’Engle K, Lanham M, Loolpapit M, Oguma I. Understanding partial protection and HIV risk and behavior following voluntary medical male circumcision rollout in Kenya. Health Educ Res 2014; 29: 122–30. 24293524 10.1093/her/cyt103 PMC3894669

[62] Riess TH, Achieng’ MM, Otieno S, Ndinya-Achola JO, Bailey RC. “When I was circumcised I was taught certain things”: risk compensation and protective sexual behavior among circumcised men in Kisumu, Kenya. PLoS One 2010; 5: e12366. 20811622 10.1371/journal.pone.0012366 PMC2928269

[63] Limburgh CM, van Schalkwyk GI, Lee KH, Cutting to the chase: participation factors, behavioral effects, and cultural perspectives of participants in an adult circumcision campaign. AIDS Care 2013; 25: 1278–83. 23383709 10.1080/09540121.2013.764392

[64] Grund JM, Hennink MM. A qualitative study of sexual behavior change and risk compensation following adult male circumcision in urban Swaziland. AIDS Care 2012; 24: 245–51. 21777079 10.1080/09540121.2011.596516

[65] Kibira SPS, Atuyambe LM, Sandøy IF, Makumbi FE, Daniel M. “Now that you are circumcised, you cannot have first sex with your wife”: post circumcision sexual behaviours and beliefs among men in Wakiso district, Uganda. J Int AIDS Soc 2017; 20: 21498. 28605174 10.7448/IAS.20.1.21498 PMC5515054

[66] Kelly A, Kupul M, Fitzgerald L, “Now we are in a different time; various bad diseases have come.” Understanding men’s acceptability of male circumcision for HIV prevention in a moderate prevalence setting. BMC Public Health 2012; 12: 67. 22264256 10.1186/1471-2458-12-67 PMC3298502

[67] Ledikwe JH, Mawandia S, Kleinman NJ, Voluntary medical male circumcision and perceived sexual functioning, satisfaction, and risk behavior: a qualitative study in Botswana. Arch Sex Behav 2020; 49: 983–98. 31997131 10.1007/s10508-019-01589-7

[68] Haberland NA, Kelly CA, Mulenga DM, Mensch BS, Hewett PC. Women’s perceptions and misperceptions of male circumcision: a mixed methods study in Zambia. PLoS One 2016; 11: e0149517. 26937971 10.1371/journal.pone.0149517 PMC4777382

[69] Shi CF, Li M, Dushoff J. Evidence that promotion of male circumcision did not lead to sexual risk compensation in prioritized sub-Saharan countries. PLoS One 2017; 12: e0175928. 28441458 10.1371/journal.pone.0175928 PMC5404849

[70] Centre for Evidence-Based Medicine. Oxford Centre for Evidence-Based Medicine: levels of evidence. March, 2009. https://www.cebm.net/2009/06/oxford-centre-evidence-based-medicine-levels-evidence-march-2009/ (accessed Nov 16, 2020).

[71] Alsallaq RA, Cash B, Weiss HA, Quantitative assessment of the role of male circumcision in HIV epidemiology at the population level. Epidemics 2009; 1: 139–52. 21352761 10.1016/j.epidem.2009.08.001

[72] Andersson KM, Owens DK, Paltiel AD. Scaling up circumcision programs in southern Africa: the potential impact of gender disparities and changes in condom use behaviors on heterosexual HIV transmission. AIDS Behav 2011; 15: 938–48. 20924783 10.1007/s10461-010-9784-y PMC3112296

[73] Fisher RJ. Social desirability bias and the validity of indirect questioning. J Consum Res 1993; 20: 303–15.

