# Supplementary material for: Association between medical male circumcision and HIV risk compensation among heterosexual men: a systematic review and meta-analysis
Source: Lancet Glob Health. Author manuscript; Available in PMC 2021 Dec 21. (PMC8690595; doi:10.1016/S2214-109X(21)00102-9)
Supplement: 1 [file NIHMS1715711-supplement-1.pdf]

THE LANCET
Global Health

Supplementary appendix

This appendix formed part of the original submission and has been peer reviewed. We post it as supplied by the authors.

Supplement to: Gao Y, Yuan T, Zhan Y, et al. Association between medical male circumcision and HIV risk compensation among heterosexual men: a systematic review and meta-analysis. *Lancet Glob Health* 2021; published online April 30. http://dx.doi.org/10.1016/S2214-109X(21)00102-9.

Supplementary files

Abbreviations used in the supplementary files 2

Supplementary Text. Further details on methods 3

Supplementary Text 1. Data extraction 3

Supplementary Text 2. Quality assessment scale 5

Supplementary Text 3. Data analysis 10

Supplementary Table S1. Characteristics of included studies examining the association between MMC and condomless sex among heterosexual men 11

Supplementary Table S2. Characteristics of included studies examining the association between MMC and multiple sex partners among heterosexual men 16

Supplementary Table S3. Univariate meta-regression analysis of the associations between MMC and condomless sex and multiple sex partners 20

Supplementary Table S4. Risk of bias assessment for cross-sectional studies – Newcastle-Ottawa Scale (adaptation) 21

Supplementary Table S5. Risk of bias assessment for cohort studies – Newcastle-Ottawa Scale (adaptation) 22

Supplementary Table S6. Risk of bias assessment for interventional studies - Cochrane Collaboration's tool 23

Supplementary Table S7. Subgroup meta-analyses of studies conducted after 2007 of the association between MMC and HIV risk compensation among heterosexual men 24

Supplementary Figure S1. Sensitivity analysis of OR estimates on the association between MMC and condomless sex among heterosexual men 25

Supplementary Figure S2. Sensitivity analysis of OR estimates on the association between MMC and multiple sex partners among heterosexual men 26

Supplementary Figure S3. Funnel plot of the log OR estimates of the association between MMC and condomless sex among heterosexual men 27

Supplementary Figure S4. Funnel plot of the log OR estimates of the association between MMC and multiple sex partners among heterosexual men 28

References: 29

Abbreviations used in the supplementary files

CI = Confidence interval

LCB = The lower 95% CI bound of OR

MMC = Medical male circumcision

OR = Odds ratio

RCT = Randomized controlled trial

RR = Relative risk

SE = Standard error

UCB = The upper 95% CI bound of OR

WHO = World Health Organization

Supplementary Text. Further details on methods

Supplementary Text 1. Data extraction

1.1 Because we used ORs as association estimates in meta-analyses, we extracted ORs/RRs of no condom use/multiple sex partner comparing circumcised and uncircumcised male from included studies, with adjusted ORs/RRs extracted preferentially over unadjusted ORs/RRs.

1.2 If a study did not directly report ORs/RRs and/or 95% CIs, we extracted required data to calculate crude ORs and 95% CIs.

1.3 If cohort studies reported both baseline cross-sectional data and prospective incident data, incident data were preferentially extracted.

1.4 If the studies reported condom use consistency, last one-time condom use and unprotected sexual intercourse, we prioritized the extraction the condom use consistency data, followed by the unprotected sexual intercourse, and finally the last one-time condom use.

1.5 Some studies had applied a categorical variable of 3 levels, i.e., consistent condom use, inconsistent condom use, and no condom use. Other studies reported condom use behavior in a binary format i.e. unprotected intercourse in the previous several months or condom use in last one-time sex or consistent condom use and inconsistent condom use. For the purpose of consistency, the outcome variable was reconstructed in terms of reported condom use during any sexual activity. The reported condom use that was categorized as consistent, inconsistent and no use condom, while inconsistent condom use and no condom use were combined to one group, and consistent condom use as the other group.

1.6 If studies reported that there were any partners and non-marriage partners in the heterosexuals, we preferentially extracted data of any partner. When there was a contradiction between partner type and condom use, the principle of priority 1.4.

1.7 If studies reported the number of sex partners in different approach, we preferentially extracted data that could be classified by ≤ 1 and ≥ 2. It was defined as multiple sex partners that the number of sex partners ≥ 2. Some studies reported the number of any type sex partner while others reported the number of non-marriage partner. The number of sex partner was reset in a binary format i.e. ≥ 2 and ≤ 1, no matter any partner or non-marriage partner or casual partner.

1.8 If studies reported marital status including married, married (not living with wife), married (living with wife), not married (no live-in partner), not married (with live-in partner), single, separated, divorced widowed and previously married, we redefined the married (living with wife), married and not married (with live-in partner) as married/cohabiting.

1.9 We extracted both the overall sample size and analytical sample of condomless sex and/or multiple sex partners. The analytical sample was used to compute association estimates between circumcision and condomless sex and multiple sex partners. Discrepancies between the analytical sample and overall sample size were due to missing data. For example, if a study recruited 1000 male and examined their circumcision status, but only 800 participants completed questionnaire survey or follow up visit, and 600 participants provided information data about condom use and/or reported their number of sex partner, then the overall sample size, observation/follow-up sample size, analytical sample of condom use and/or number of sex partner were 1000, 800, and 600, respectively.

1.10 If studies reported both median age and mean age, mean age was preferentially extracted.

Supplementary Text 2. Quality assessment scale

Newcastle-Ottawa Scale adapted for cross-sectional studies

A. Selection: (Maximum 4 points)

1) Representativeness of the sample

a) Representative of participant (score 1 point if one of the following items was fulfilled).

(1) Non-clinic-based sample: the study employed respondent driven sampling/mixed methods/venue-time-space sampling to recruit male from different sites.

(2) The study reanalyzed data from National Systems or randomized clinical trials.

b) Selected group of participants (score 0 point if one of the following items were fulfilled)

(1) Clinic-based sample.

(2) The study recruited male from only one site using convenience sampling.

c) No description of the derivation of the sample (score 0 point).

2) Sample size

a) Pre-determined and satisfactory (score 1 point).

b) Not pre-calculated (score 0 point).

3) Non-respondents

a) Score 1 point one of the following items were fulfilled:

(1) Comparability between respondent and non-respondent characteristics is established, and the response rate is satisfactory.

(2) Response rate =100%.

(3) The study employed multiple imputation approach to account for the uncertainty in missing responses.

b) The response rate is unsatisfactory, or the comparability between respondents and non-respondents is unsatisfactory (score 0 point).

c) No description of the response rate or the characteristics of the respondents and the non-respondents (score 0 point).

4) Ascertainment of circumcision status

a) Physical examination/medical record (score 1 point).

b) Self-reported (score 0 point).

c) No description (score 0 point).

B. Comparability (Maximum 2 points)

1) Study controlled for factors that lead to sexual risk behavior, such as alcohol consumption, recreational drugs use (score 1 point).

2) Study controlled for other demographic characteristics (score 1 point).

C. Outcome (Maximum 3 points)

1) Assessment of the number of sex partner and condom use status

a) Self-reported (score 1 point).

b) Other / no description (score 0 point).

2) Statistical test

a) The statistical test used to analyze the data is clearly described and appropriate, and the measurement of the association is presented, including confidence intervals (score 1 point).

b) The statistical test is not appropriate, not described or incomplete (score 0 point).

Newcastle-Ottawa scale for cohort studies (adapted version)

A. Selection (Maximum 4 points)

1) Representatives of the sample

a) Representative of participant (score 1 point if one of the following items were fulfilled).

(1) Non-clinic-based sample: the study employed respondent driven sampling/mixed methods/venue-time-space sampling to recruit male from different sites.

(2) The study reanalyzed data from National Systems or randomized clinical trials.

b) Selected group of participants (score 0 point if one of the following items were fulfilled)

(1) Clinic-based sample.

(2) The study recruited male from only one site using convenience sampling.

c) No description of the derivation of the sample.

2) Selection of the non-intervention cohort

a) Drawn from the same source of population (score 1 point).

b) Drawn from a different source/No description (score 0 point).

3) Ascertainment of circumcision status

a) Physical examination (score 1 point).

b) Self-reported/ No description (score 0 point).

4) Demonstration that outcome of interest was not present at start of study

a) Yes (score 1 point).

b) No/unclear (score 0 point).

B. Comparability (Maximum 2 points)

1) Study controlled for factors that lead to sexual risk behavior, such as alcohol consumption, recreational drugs use (score 1 point).

2) Study controlled for other demographic characteristics (score 1 point).

C. Outcome (Maximum 4 points)

1) Assessment of the number of sex partner and condom use status

a) Self-reported (score 1 point).

b) Other / no description (score 0 point).

2) Was follow up long enough for outcomes to occur?

a) Yes, if median duration of follow-up ≥ 6 months (score 1 point).

b) No, if median duration of follow-up < 6 months (score 0 point).

3) Adequacy of follow up of cohorts

a) Complete follow up: all subjects accounted for (score 1 point).

b) Subjects lost to follow up unlikely to introduce bias: number lost ≤ 20%, or description of those lost suggesting no different from those followed (score 0 point).

Cochrane Collaboration's tool for assessing risk of bias of intervention studies

(A=low risk of bias, B=high risk of bias, C=unclear)

Sequence generation

A: investigators described a random component in the sequence generation process, such as the use of random number table, coin tossing, card or envelope shuffling, etc.

B: investigators described a non-random component in the sequence generation process, such as the use of odd or even date of birth, algorithm based on the day or date of birth, hospital, or clinic record number.

C: insufficient information to permit judgement of the sequence generation process.

Allocation concealment

A: participants and the investigators enrolling participants cannot foresee assignment (e.g., central allocation; or sequentially numbered, opaque, sealed envelopes).

B: participants and investigators enrolling participants can foresee upcoming assignment (e.g., an open random allocation schedule, a list of random numbers); or envelopes were unsealed or non-opaque or not sequentially numbered.

C: insufficient information to permit judgement of the allocation concealment or the method not described.

Blinding

A: blinding of the participants, key study personnel, and outcome assessor, and unlikely that the blinding could have been broken. Or lack of blinding unlikely to introduce bias. No blinding in the situation where non-blinding is not likely to introduce bias.

B: no blinding, incomplete blinding and the outcome is likely to be influenced by lack of blinding.

C: insufficient information to permit judgement of adequacy or otherwise of the blinding.

Incomplete outcome data

A: no missing outcome data, reasons for missing outcome data unlikely to be related to true outcome, or missing outcome data balanced in number across groups.

B: reason for missing outcome data likely to be related to true outcome, with either imbalance in number across groups or reasons for missing data.

C: insufficient reporting of attrition or exclusions.

Selective reporting

A: a protocol is available which clearly states the primary outcome as the same as in the final trial report.

B: the primary outcome differs between the protocol and final trial report.

C: no trial protocol is available or there is insufficient reporting to determine if selective reporting is present.

Other bias

A: there is no evidence of bias from other sources.

B: there is potential bias present from other sources (e.g., early stopping of trial, fraudulent activity, extreme baseline imbalance, or bias related to specific study design).

C: insufficient information to permit judgement of adequacy or otherwise of other forms of bias.

Note: It is impossible to blind participants and the personnel delivering a circumcision intervention. Hence, we did not assess the risk of study bias in blinding, and only assessor blinding is considered.

Supplementary Text 3. Data analysis

3.1 Calculation of crude ORs and their 95% CIs

If a study did not directly report ORs and their 95% CIs, we first reconstructed the following fourfold table:

	No condom use/ Number of sex partner ≥ 2	Condom use/ Number of sex partner ≤ 1
Circumcised	a	b
Uncircumcised	c	d

Where a, b, c, and d, are the number of men.

We then used the following command of Stata version 15.1 to calculate crude ORs and their 95%CIs: cci a b c d, exact

3.3 Meta-analysis

We calculated pooled OR estimates and their 95% CIs with natural log-transformed ORs (logORs) and SEs, based on the DerSimonian-Laird inverse variance method.

SElog (OR)= (log (UCB)-log (LCB)) / 3·92

where UCB is the upper 95% CI bound and LCB is the lower 95% CI bound of OR.

Pooled ORs and their 95% CIs were hen back-transformed to the original scale.

Supplementary Table S1. Characteristics of included studies examining the association between MMC and condomless sex among heterosexual men

Study (country)	Enrollment period/Study design	WHO region/ income level [a]	Circumcision time	Setting/ Participant No.	Mean and/or median age of circumcision men, year	Married/ cohabiting, (%)	Circumcision ascertainment, No./ Prevalence, No. (%)	Follow up /retrospective duration [b]	Condom use ascertainment/ Analytic sample, No. [c]	Condomless		(Adjusted/ crude) OR (95%CI) [d]	Type of partner for condomless	condom use period or observation time	Risk of bias[e]: Participant selection/Comparability/Outcome or exposure
										No. Circumcised/ Total No. (%)	No. Uncircumcised/ Total No. (%)				
Kong et al. 2012[1] (Uganda)	2007-2011/ Prospective cohort	Africa/Low income	2006-2010	Non-clinic-based /2137	Not reported	996/1597 (62·4)	Genital examination, -	Baseline	self-reported/1362	961/1101 (87·3)	229/261 (87·7)	-	Any	Previous 12 months	4/2/2
							Genital examination, 1297 /1597 (81·2)	3·13 year	Self-reported /1362	1025/1101 (93·1)	242/261 (92·7)	Crude:1·06 (0·63,1·78)	Any	3·13 years	
Feldblum et al. 2015[2] (Kenya)	2012/ Prospective cohort [f]	Africa/Lower-middle income	2012	Clinic-based /199	24·8/22	-	Genital examination, 194 /388 (50·0)	32months	Self-reported /349	147/194 (75·8)	92/155 (59·4)	Crude:2·14 (1·35,3·39)	Any	32 months	3/1/3
Mukudu et al. 2019[3] (South Africa)	2012-2014/ Prospective cohort [f]	Africa/Upper-middle income	2012-2014	Clinic-based /496	25·5	-	Genital examination, 233 /466 (50·0)	12 months	Self-reported /466	121/233 (51·9)	141/233 (60·5)	Crude:0·70 (0·49,1·02)	Any	last time	3/2/3
Agot et al. 2007[4](Kenya)	2002-2004/ Prospective cohort	Africa/Lower-middle income	2002-2004	Non-clinic-based /648	22·3	134/648 (20·1)	Genital examination, -	Baseline	Self-reported /648	141/324 (43·5)	152/324 (46·9)	-	Any	Not reported	4/1/3
							Genital examination, 187/375 (49·9)	6 months	Self-reported /375	143/187 (76·5)	138/188 (73·4)	Crude:1·18 (0·74,1·89)	Non-marriage	6 months	
							Genital examination, 193/374 (51·6)	9 months	Self-reported /374	146/193 (75·6)	138/181 (76·2)	Crude:0·97 (0·60,1·56)	Non-marriage	9 months	
							Genital examination, 165/350 (47·1)	12 months	Self-reported /350	115/165 (69·7)	133/185 (71·9)	Crude:0·92 (0·58,1·46)	Non-marriage	12 months	
Bailey et al. 2007[5] (Kenya)	2002-2005/ RCT	Africa/Lower-middle income	2002-2005	Non-clinic-based /2784	20·0	156/2773 (5·6)	Genital examination, -	Baseline	Self-reported /2386	928/1193 (77·8)	939/1193 (78·7)	-	Any	Previous 6 months	Low
							Genital examination, 1040/2086 (49·9)	6 months	Self-reported /2086	670/1040 (64·4)	668/1046 (63·9)	Crude:1·02 (0·85,1·22)	Any	6 months	
							Genital examination,	12 months	Self-reported	681/1039	627/1025	Crude:1·20	Any	12 months	

							1039/2064 (50·3)		/2064	(65·5)	(61·2)	(1·01,1·45)			
							Genital examination, 830/1652 (50·2)	18 months	Self-reported /1652	534/830 (64·3)	518/822 (63·0)	Crude:1·06 (0·87,1·30)	Any	18 months	
							Genital examination, 637/1232 (51·7)	24 months	Self-reported /1232	406/637 (63·7)	349/595 (58·7)	Crude:1·24 (0·98,1·56)	Any	24 months	
Gray et al. 2007[6] (Uganda)	2004-2006/ RCT	Africa/Low income	2004-2006	Non-clinic-based /4996	Not reported	2340/4996 (46·8)	Genital examination, -	Baseline	Self-reported /4034	1667/2006 (83·1)	1673/2028 (82·5)	-	Any	Previous 12 months	Low
							Genital examination, 1801/3588 (50·2)	6 months	Self-reported /3588	1467/1801 (81·5)	1492/1787 (83·5)	Crude:0·87 (0·73,1·03)	Any	6 months	
							Genital examination, 1815/3588 (50·6)	12 months	Self-reported /3588	1482/1815 (81·7)	1450/1773 (81·8)	Crude:0·99 (0·84,1·18)	Any	12 months	
							Genital examination, 846/1696 (49·9)	24 months	Self-reported /1696	688/846 (81·3)	690/850 (81·2)	Crude:1·01 (0·79,1·29)	Any	24 months	
Westercamp et al. 2014[7] (Kenya)	2008-2010/ Prospective cohort	Africa/Lower-middle income	2008-2010	Non-clinic-based /3186	20·0	1095/3186 (34·4)	Genital examination, -	Baseline	Self-reported /2570	693/1263 (53·0)	672/1307 (51·4)	-	Any	last time	4/0/2
							Genital examination, 1263/2355 (53·6)	6 months	Self-reported /2355	810/1263 (64·1)	522/1092 (47·8)	Crude:1·96 (1·67,2·33)	Any	last time	
							Genital examination, 1151/2308 (49·9)	12 months	Self-reported /2308	480/1151 (41·7)	556/1157 (48·1)	Crude:0·78 (0·66,0·91)	Any	last time	
							Genital examination, 1321/2425 (54·5)	18 months	Self-reported /2425	518/1321 (39·2)	509/1104 (46·1)	Crude:0·75 (0·64,0·78)	Any	last time	
							Genital examination, 1468/2517 (58·3)	24 months	Self-reported /2517	571/1468 (38·9)	506/1049 (48·2)	Crude:0·68 (0·58,0·80)	Any	last time	
Kagaayi et al. 2016[8] (Uganda)	2008-2011/ Retrospective cohort	Africa/Low income	2006-2009	Non-clinic-based /5494	26·1	2787/4907 (56·8)	Genital examination, -	Baseline	Self-reported /1955	113/261 (43·3)	727/1694 (42·9)	-	Any	last time	4/1/3
							Genital examination, 578/4907 (11·8)	18 months	Self-reported /1954	101/251 (40·2)	763/1703 (44·8)	Crude :0·83 (0·63,1·09)	Non-marriage	last time	
Govender et al.	2011-2013/	Africa/Upper-	2011-2013	Non-clinic-	17·3	-	Genital examination, -	Baseline	Self-reported	64/194	69/253	-	Any	Not	4/0/2

2018[9] (South Africa)	Retrospective cohort	middle income		based /981					/447	(33·0)	(27·3)			reported	
							Genital examination, 393/755 (52·1)	6 months	Self-reported /423	53/225 (23·6)	49/198 (24·7)	Crude:0·93 (0·60,1·47)	Any	6 months	
							Genital examination, 420/858 (49·0)	12 months	Self-reported /523	45/232 (19·4)	52/291 (17·9)	Crude:1·11 (0·71,1·72)	Any	12 months	
Brito et al. 2017[10] (Dominican)	2013-2014/ Prospective cohort [f]	America/Upper-middle income	2013-2014	Non-clinic-based /454	26·0	-	Genital examination, 317 /634 (50·0)	6-24months	Self-reported /634	192/316 (60·8)	182/317 (57·4)	Crude:1·15 (0·84,1·58)	Any	last time	2/2/3
Tarnaud et al 2011[11] (South Africa)	2002-2004/ RCT	Africa/Upper-middle income	2002-2004	Non-clinic-based /1753	21·2	47/934(5·0)	Genital examination, 890/1753 (50·8)	21 months	Self-reported /934	373/497 (73·0)	334/437 (76·4)	Crude:0·93 (0·69,1·25)	Any	12 months	Low
Bailey et al. 1999[12] (Uganda)	1997/Cross-sectional	Africa/Low income	Not reported	Non-clinic-based /365	30·8	-	Not reported, 144/320 (45·0)	last time	Self-reported /320	125/144 (86·8)	155/176 (88·1)	Crude :0·89 (0·46,1·73)	Non-marriage	last time	1/2/2
Ayiga et al. 2011[13] (Botswana)	2008/ Cross-sectional	Africa/Upper-middle income	infancy and after infancy	Non-clinic-based /1257	Not reported	-	Self-reported, 116/ 1192 (9·7)	last time	Self-reported /1192	18/116 (15·5)	126/1076 (11·7)	Crude:1·38 (0·81,2·37)	Any	last time	1/0/1
Forbes et al. 2012[14] (Tanzania)	2007-2008/ Cross-sectional [g]	Africa/Low income	1999-2002	Non-clinic-based /7300	22·0	2444/7300 (33·5)	Genital examination, 1436/3509 (40·9)	Previous 1 month	Self-reported /3509	577/1436 (40·2)	1290/2073 (62·2)	Crude :0·41 (0·36,0·47)	Non-marriage	Previous 1 month	1/0/2
Auvert et al. 2013[15] (South Africa)	2007-2008/ Cross-sectional [g]	Africa/Upper-middle income	2007-2008	Non-clinic-based /1998	Not reported	262/1988 (13·2)	Genital examination, 329/1988 (16·5)	Previous 12 months	Self-reported /1474	169/272 (62·1)	648/1202 (53·9)	Crude:1·40 (1·07,1·84)	Non-marriage	Previous 12 months	2/0/2
	2010 - 2011/ Cross-sectional [g]		2008-2010	Non-clinic-based /3338	24·5	803/3338 (24·1)	Genital examination, 1531/2678 (57·2)	Previous 12 months	Self-reported /2678	873/1531 (57·0)	644/1147 (56·1)	Crude:1·04 (0·89,1·21)	Non-marriage	Previous 12 months	
Galbraith et al. 2014[16] (Kenya)	2012/ Cross-sectional	Africa/Lower-middle income	Not reported	Non-clinic-based /874	Not reported	261/847 (30·8)	Self-reported, 435/874 (49·8)	Previous 12 months	Self-reported /415	90/153 (58·8)	220/262 (84·0)	Crude:0·27 (0·17,0·43)	Any	Previous 12 months	1/0/1
Balekang et al. 2016[17] (Botswana)	2008/ Cross-sectional	Africa/Upper-middle income	Not reported	Non-clinic-based /313	Not reported	251/301 (83·4)	Not reported, 53/313 (16·9)	Previous 12 months	Self-reported /229	15/42 (35·7)	54/187 (28·9)	Crude:1·37 (0·68,2·77)	Any	Previous 12 months	1/0/1
Kibira et al. 2016[18] (Uganda)	2004/ Cross-sectional	Africa/Low income	Not reported	Non-clinic-based /9905	Not reported	4621/6906 (66·9)	Self-reported, 1792/6906 (25·9)	last time	Self-reported /2039	290/636 (45·6)	692/1403 (49·3)	Crude :0·86 (0·71,1·04)	Non-marriage	last time	1/0/2
	2011/ Cross-sectional		Not reported	Non-clinic-based /9983	Not reported	5710/7969 (71·7)	Self-reported, 2228/7969 (28·0)	last time	Self-reported /2315	448/768 (58·3)	819/1547 (52·9)	Crude :1·24 (1·04,1·48)	Non-marriage	last time	

Zungu et al. 2016[19] (South Africa)	2012/ Cross-sectional	Africa/Upper-middle income	Not reported	Non-clinic-based /8240	Not reported	-	Self-reported, 2121/8240 (25·7)	last time	Self-reported /4961	944/1440 (65·6)	2380/3521 (67·6)	Crude:0·91 (0·80,1·04)	Any	last time	1/0/2
George et al. 2017[20] (South Africa)	2012-2013/ Cross-sectional	Africa/Upper-middle income	2011-2013	Non-clinic-based /750	16·8	-	Genital examination, 251/750 (33·5)	Previous 1 month	Self-reported /750	148/251 (59·0)	309/499 (61·9)	Crude:0·88 (0·65,1·20)	Any	Previous 1 month	2/0/1
Westercamp et al. 2017[21] (Kenya)	2008-2009/ Cross-sectional	Africa/Lower-middle income	Not reported	Non-clinic-based /1210	24·0	326/675 (48·3)	Self-reported, 215/675 (31·9)	Previous 12 months	Self-reported /525	113/187 (60·4)	215/338 (63·5)	Crude:0·87 (0·60,1·26)	Non-marriage	Previous 12 months	1/0/1
	2011/ Cross-sectional			Non-clinic-based /1540	26·0	823/1371 (60·0)	Self-reported, 669/1371 (48·8)	Previous 12 months	Self-reported /967	260/456 (57·0)	326/511 (63·7)	Crude:0·75 (0·58,0·98)	Non-marriage	Previous 12 months	
	2013/ Cross-sectional			Non-clinic-based /1442	27·0	741/1308 (56·7)	Self-reported, 781/1308 (59·7)	Previous 12 months	Self-reported /955	233/551 (42·2)	177/404 (43·7)	Crude:0·94 (0·73,1·22)	Non-marriage	Previous 12 months	
Ortblad, et al. 2019[22] (South Africa)	2009-2015/ Cross-sectional [g]	Africa/Upper-middle income	Not reported	Non-clinic-based /5127	17·0	-	Self-reported, 1235/5127 (24·1)	last time	Self-reported /5127	110/1235 (8·9)	552/3892 (14·2)	Crude:0·59 (0·48,0·73)	Any	last time	2/0/2
Kufa et al. 2020[23] (South Africa)	2017-2018/ Cross-sectional	Africa/Upper-middle income	Not reported	Non-clinic-based /750	27·0	-	Genital examination, 166/496 (33·5)	last time	Self-reported /496	137/166 (82·5)	271/330 (82·1)	Crude:1·03 (0·63,1·68)	Any	last time	3/1/2
Wei et al. 2018[24] (China)	2004/ Cross-sectional [g]	Western Pacific/Upper-middle income	Not reported	Non-clinic-based /673	28·0	499/673 (74·1)	Genital examination, 113/673 (16·8)	Not reported	Self-reported /673	100/113 (88·5)	511/560 (91·2)	Crude:0·74 (0·39,1·41)	Any	Not reported	4/1/1
Mwandi et al. 2012[25] (Kenya)	2007/ Cross-sectional	Africa/Lower-middle income	Not reported	Non-clinic-based /8883	32·0	4469/7678 (58·2)	Genital examination, 6586/7678 (85·8)	Previous 12 months	Self-reported /5747	4389/4990 (88·0)	633/757 (83·6)	Crude:1·43 (1·16,1·77)	Any	Previous 12 months	3/1/1
Reed et al. 2012[26] (Swaziland)	2010-2011/ Cross-sectional	Africa/Lower-middle income	2009	Non-clinic-based /7075	21·1	-	Genital examination, 1105/7075 (15·6)	Previous 6 months	Self-reported /7075	674/1105 (61·0)	4000/5970 (67·0)	Crude:0·77 (0·67,0·88)	Any	Previous 6 months	3/1/1

[a] Studies grouped by country in which the study was conducted.

[b] Prospective period and retrospective period were used in cohort, and retrospective period was used in cross-sectional study.

[c] The analytic sample was used to calculate the association between circumcision and condomless sex and multiple sex partner. Discrepancies between analytic samples and overall sample size were due to missing data.

[d] ORs were extracted directly from articles were available, with adjusted odds ratios extracted preferentially over unadjusted odds ratios. Crude ORs were calculated based on reconstructed fourfold tables if they were not reported.

[e] The adapted version of the Newcastle-Ottawa Scale was used to assess risk of bias of studies along three aspects: participant selection (4 criteria), comparability between study groups (1 criterion), and assessment of outcome or exposure (3 criteria for cohort, 2 criteria for cross-sectional studies).

[f] Study did not have a control group at baseline, and comparison before and after follow-up.

[g] Cross-sectional information from cohort.

A total of 26 studies reported condom use status.

Supplementary Table S2. Characteristics of included studies examining the association between MMC and multiple sex partners among heterosexual men

Study (country)	Enrollment period/Study design	WHO region/Income Level [a]	Circumcision time	Setting/ Participant No.	Mean or median age, year	Married/ cohabiting, (%)	Circumcision ascertainment, No. /Prevalence, No. (%)	Follow up /retrospective duration [b]	Number of sex partner ascertainment / Analytic sample, No. [c]	Number of sex partners≥2		(Adjusted/ crude) OR (95%CI) [d]	Type of sexual partner	Risk of bias[e]: Participant selection/Comparability/Outcome or exposure
										No. Circumcised /Total No. (%)	No. Uncircumcised /Total No. (%)			
Kong et al. 2012[1] (Uganda)	2007-2011/ Prospective cohort	Africa/Low income	2006-2010	Non-clinic-based /2137	Not reported	996/1597 (62·4)	Genital examination, -	Baseline	Self-reported /1597	444/1297 (34·2)	101/300 (33·7)	-	Any	4/2/2
							Genital examination, 1297/1597 (81·2)	3·13 year	Self-reported /1597	468/1297 (36·1)	126/300 (42·0)	Crude:0·78 (0·60,1·01)	Any	
Feldblum et al. 2015[2] (Kenya)	2012/ Prospective cohort [f]	Africa/Lower-middle income	2012	Clinic-based /199	24·8	-	Genital examination, 194/388 (50·0)	32 months	Self-reported /349	82/194 (42·3)	45/155 (29·0)	Crude:1·79 (1·14,2·80)	Any	3/1/3
Mukudu et al. 2019[3] (South Africa)	2012-2014/ Prospective cohort [f]	Africa/Upper-middle income	2012-2014	Clinic-based /496	Not reported	-	Genital examination, 233/466 (50·0)	12 months	Self-reported /466	71/233 (30·5)	70/233 (30·0)	Crude:1·02 (0·69,1·52)	Any	3/2/3
Agot et al. 2007[4] (Kenya)	2002-2004/ Prospective cohort	Africa/Lower-middle income	2002-2004	Non-clinic-based /648	22·3	134/648 (20·7)	Genital examination, -	Baseline	Self-reported /648	20/324 (6·2)	15/324 (4·6)	-	Any	4/1/3
							Genital examination, 298/579 (51·5)	6 months	Self-reported /579	9/298 (3·0)	11/281 (3·9)	Crude:0·76 (0·31,1·89)	Non-marriage	
							Genital examination, 291/561 (51·9)	9 months	Self-reported /561	5/291 (1·7)	8/270 (3·0)	Crude:0·57 (0·18,1·79)	Non-marriage	
							Genital examination, 288/557 (51·7)	12 months	Self-reported /557	5/288 (1·7)	7/269 (2·6)	Crude:0·66 (0·21,2·11)	Non-marriage	
Bailey et al. 2007[5] (Kenya)	2002-2005/ RCT	Africa/Lower-middle income	2002-2005	Non-clinic-based /2784	20·0	156/2773 (5·6)	Genital examination, -	Baseline	Self-reported /2777	585/1388 (42·1)	579/1389 (41·7)	-	Any	low
							Genital examination, 1232/2495 (49·4)	6 months	Self-reported /2495	409/1232 (33·2)	443/1263 (35·1)	Crude:0·92 (0·78,1·09)	Any	
							Genital examination, 1227/2455 (50·0)	12 months	Self-reported /2456	360/1227 (29·3)	408/1229 (33·2)	Crude:0·83 (0·71,0·99)	Any	

							Genital examination, 985/1973 (49·9)	18 months	Self-reported /1973	294/985 (29·8)	300/988 (30·4)	Crude:0·98 (0·81,1·18)	Any	
							Genital examination, 741/1469 (50·4)	24 months	Self-reported /1469	225/741 (30·4)	199/728 (27·3)	Crude:1·16 (0·92,1·45)	Any	
Gray et al. 2007[6] (Uganda)	2004-2006/ RCT	Africa/Low income	2004-2006	Non-clinic-based /4996	Not reported	2340/4996 (46·8)	Genital examination, -	Baseline	Self-reported /4996	854/2474 (34·5)	860/2522 (34·1)	-	Any	low
							Genital examination, 2268/4589 (49·4)	6 months	Self-reported /4589	538/2268 (23·7)	564/2321 (24·3)	Crude:0·97 (0·85,1·11)	Any	
							Genital examination, 2253/4503 (50·0)	12 months	Self-reported /4502	566/2252 (25·1)	572/2250 (25·4)	Crude:0·98 (0·86,1·12)	Any	
							Genital examination, 978/1973 (49·6)	24 months	Self-reported /1972	347/977 (35·5)	352/995 (35·4)	Crude:1·01 (0·84,1·21)	Any	
Westercamp et al. 2014[7] (Kenya)	2008-2010/ Prospective cohort	Africa/Lower-middle income	2008-2010	Non-clinic-based /3186	20·0	1095/3186 (34·4)	Genital examination, -	Baseline	Self-reported /1807	500/873 (57·3)	551/934 (59·0)	-	Any	4/0/2
							Genital examination, 644/1471 (43·8)	6 months	Self-reported /1471	279/644 (43·3)	373/827 (45·1)	Crude:0·93 (0·76,1·15)	Any	
							Genital examination, 858/1737 (49·4)	12 months	Self-reported /1737	333/858 (38·8)	353/879 (40·2)	Crude:0·94 (0·78,1·15)	Any	
							Genital examination, 1034/1885 (54·9)	18 months	Self-reported /1885	363/1034 (35·1)	332/851 (39·0)	Crude:0·85 (0·70,1·02)	Any	
							Genital examination, 1177/2020 (58·3)	24 months	Self-reported /2020	371/1177 (31·5)	281/843 (33·3)	Crude:0·95 (0·79,1·15)	Any	
Kagaayi et al. 2016[8] (Uganda)	2008-2011/ Retrospective cohort	Africa/Low income	2006-2009	Non-clinic-based /5494	26·1	2787/4907 (56·8)	Genital examination, -	Baseline	Self-reported /3835	208/431 (48·3)	1416/3404 (41·6)	-	any	4/1/3
							Genital examination, 578/4907 (11·8)	18 months	Self-reported /4015	196/453 (43·3)	1381/3562 (38·8)	Crude :1·20 (0·99,1·47)	Any	
Govender et al. 2018[9] (South Africa)	2011-2013/ Retrospective cohort	Africa/Upper-middle income	2011-2013	Non-clinic-based /981	17·3	-	Genital examination, -	Baseline	Self-reported /449	44/195 (22·6)	41/254 (16·1)	-	Any	4/0/2
							Genital examination,	6 months	Self-reported	32/225	44/198	Crude:0·58	Any	

							393/755 (52·1)		/423	(14·2)	(22·2)	(0·35,0·96)		
							Genital examination, 420/858 (49·0)	12 months	Self-reported /523	49/232 (21·1)	66/291 (22·7)	Crude:0·91 (0·60,1·39)	Any	
Brito et al. 2017[10] (Dominican)	2013-2014/ Prospective cohort [f]	America/Upper -middle income	2013-2014	Non-clinic-based /454	26·0	-	Genital examination, 317/634 (50·0)	6-24months	Self-reported /634	187/317 (59·0)	190/317 (59·9)	Crude:0·96 (0·70,1·32)	Any	2/2/3
Bailey et al. 1999[12] (Uganda)	1997/ Cross-sectional	Africa/Low income	Not reported	Non-clinic-based /365	30·8	-	Not reported, 188/365 (51·5)	life time	Self-reported /365	21/188 (11·2)	11/177 (6·2)	Crude:1·90 (0·89,4·06)	Any	1/2/2
Frisch et al. 2011[27] (Denmark)	2005/ Cross-sectional	Europe/High income	Not reported	Non-clinic-based /5395	Not reported	1453/2345 (62·0)	Self-reported, 125/2345 (5·3)	life time	Self-reported /2345	96/125 (76·8)	1791/2220 (80·7)	Crude:0·79 (0·52,1·22)	Any	1/0/1
Forbes et al. 2012[14] (Tanzania)	2007-2008/ Cross-sectional [g]	Africa/Low income	1999-2002	Non-clinic-based /7300	22·0	2444/7300 (33·5)	Genital examination, 2252/5698 (39·5)	Previous 1 month	Self-reported /5698	317/2252 (14·1)	569/3446 (16·5)	Crude :0·83 (0·71,0·96)	Any	1/0/2
Auvert et al. 2013[15] (South Africa)	2007-2008/ Cross-sectional [g]	Africa/Upper-middle income	2007-2008	Non-clinic-based /1998	Not reported	262/1988 (13·2)	Genital examination, 329/1988 (16·5)	Previous 12 months	Self-reported /1988	156/329 (47·4)	914/1659 (55·1)	Crude:0·74 (0·58,0·93)	Non-marriage	2/0/2
	2010 - 2011/ Cross-sectional [g]		2008-2010	Non-clinic-based /3338	24·5	803/3338 (24·1)	Genital examination, 1848/ 3338 (55·4)	Previous 12 months	Self-reported /3338	986/1848 (53·4)	744/1490 (49·9)	Crude:1·15 (1·00,1·31)	Non-marriage	
Galbraith et al. 2014[16] (Kenya)	2012/ Cross-sectional	Africa/Lower-middle income	Not reported	Non-clinic-based /874	Not reported	261/847 (30·8)	Self-reported, 435/874 (49·8)	life time	Self-reported /790	131/416 (31·5)	197/374 (52·7)	Crude:0·41 (0·31,0·55)	Any	1/0/1
Balekang et al. 2016[17] (Botswana)	2008/ Cross-sectional	Africa/Upper-middle income	Not reported	Non-clinic-based /313	Not reported	251/301 (83·4)	Not reported, 53/313 (16·9)	Previous 12 months	Self-reported /302	36/50 (72·0)	166/252 (65·9)	Crude:1·33 (0·68,2·60)	Any	1/0/1
Kibira et al. 2016[18] (Uganda)	2004/ Cross-sectional	Africa/Low income	Not reported	Non-clinic-based /9905	Not reported	4621/6909 (66·9)	Self-reported, 1792/6906 (25·9)	Previous 12 months	Self-reported /6907	592/1793 (33·0)	1118/5114 (21·9)	Crude :1·76 (1·56,1·98)	Any	1/0/2
	2011/ Cross-sectional			Non-clinic-based /9983	Not reported	5710/7969 (71·7)	Self-reported, 2228/7969 (28·0)	Previous 12 months	Self-reported /7968	613/2228 (27·5)	1168/5740 (20·3)	Crude :1·49 (1·33,1·66)	Any	
Zungu et al. 2016[19] (South Africa)	2012/ Cross-sectional	Africa/Upper-middle income	Not reported	Non-clinic-based /8240	Not reported	-	Self-reported, 2121/8240 (25·7)	Previous 12 months	Self-reported /8240	271/2121 (12·8)	504/6119 (8·2)	Crude:1·63 (1·40,1·91)	Any	1/0/2
Westercamp et al. 2017[21] (Kenya)	2008-2009/ Cross-sectional	Africa/Lower-middle income	Not reported	Non-clinic-based /1210	24·0	326/675 (48·3)	Self-reported, 215/675 (31·9)	Previous 12 months	Self-reported /675	58/215 (23·0)	99/460 (21·5)	Crude:1·35 (0·93,1·96)	Any	1/0/1
	2011/ Cross-sectional		Not reported	Non-clinic-based /1540	26·0	823/1371 (60·0)	Self-reported, 669/1371 (48·8)	Previous 12 months	Self-reported /1371	81/669 (12·1)	98/702 (14·0)	Crude:0·85 (0·62,1·16)	Any	

	2013/ Cross-sectional		Not reported	Non-clinic-based /1442	27·0	741/1308 (56·7)	Self-reported, 781/1308 (59·7)	Previous 12 months	Self-reported /1308	141/781 (18·1)	75/527 (14·2)	Crude:1·33 (0·98,1·80)	Any	
Ortblad, et al. 2019[22] (South Africa)	2009-2015/ Cross-sectional [g]	Africa/Upper-middle income	Not reported	Non-clinic-based /5127	17·0	--	Self-reported, 1235/5127 (24·1)	Previous 12 months	Self-reported /5127	65/1235 (5·3)	245/3892 (6·3)	Crude:0·83 (0·62,1·10)	Any	2/0/2
Kufa et al. 2020[23] (South Africa)	2017-2018/ Cross-sectional	Africa/Upper-middle income	Not reported	Non-clinic-based /750	27·0	-	Genital examination, 166/496 (33·5)	Previous 3 months	Self-reported /496	89/166 (53·6)	169/330 (51·1)	Crude:1·10 (0·76,1·60)	Any	3/1/2
Wei et al. 2018[24] (China)	2004/ Cross-sectional [g]	Western Pacific / Upper-middle income	Not reported	Non-clinic-based /673	28·0	499/673 (74·1)	Genital examination, 113/673 (16·8)	Not reported	Self-reported /673	15/113 (13·3)	72/560 (12·9)	Crude:0·74 (0·39,1·41)	Any	4/1/1
Mwandi et al. 2012[25] (Kenya)	2007/ Cross-sectional	Africa/Lower-middle income	Not reported	Non-clinic-based /8883	32·0	4469/7678 (58·2)	Genital examination, 6586/7678 (85·8)	Previous 12 months	Self-reported /5747	427/4426 (9·6)	121/680 (17·8)	Crude:0·49 (0·40,0·61)	Any	3/1/1

[a] Studies were grouped by country in which the study was conducted.

[b] Prospective period and retrospective period were used in cohort, and retrospective period was used in cross-sectional study.

[c] The analytic sample was used to calculate the association between circumcision and condom use and multiple sex partner. Discrepancies between analytic samples and overall sample size were due to missing data.

[d] ORs were extracted directly from articles were available, with adjusted ORs extracted preferentially over unadjusted odds ratios. Crude ORs were calculated based on reconstructed fourfold tables if they were not reported.

[e] The adapted version of the Newcastle-Ottawa Scale was used to assess risk of bias of studies along three aspects: participant selection (4 criteria), comparability between study groups (1 criterion), and assessment of outcome or exposure (3 criteria for cohort, 2 criteria for cross-sectional studies).

[f] Study did not have a control group at baseline, and comparison before and after follow-up.

[g] Cross-sectional information from cohort.

A total of 23 studies reported the number of sex partners (≥2 vs. ≤1).

Supplementary Table S3. Univariate meta-regression analysis of the associations between MMC and condomless sex and multiple sex partners

Variable	Condomless sex				Multiple sex partners			
	k [a], No.	*P* value	Variance explained R^2 (%) [b]	Meta-regression coefficient (95% confidence interval)	*k* [a], No.	*P* value	Variance explained R^2 (%) [b]	Meta-regression coefficient (95% confidence interval)
Study design [c]	30	0·51	0·0	-0·92 (-3·75, 1·90)	27	0·56	0·0	7·06 (-17·61, 31·72)
Recruitment setting	30	0·57	0·0	1·53 (-3·94, 7·01)	27	0·61	0·0	11·47 (-34·08, 57·02)
Recruitment time	30	0·35	0·0	-1·58 (-4·96, 1·80)	27	0·07	9·1	24·87 (-2·10, 51·84)
Method of ascertaining circumcision status [d]	28	0·14	4·5	-2·13 (-5·03, 0·77)	25	0·50	0·0	8·55 (-17·52, 34·62)
Age [e]	21	0·34	0·0	1·49 (-1·69, 4·68)	18	0·38	0·0	15·35 (-20·37, 51·06)
%Circumcision [f]	30	0·20	2·4	1·70 (-0·97, 4·38)	27	0·46	0·0	-8·74 (-32·48, 15·00)
Study quality [g]	30	0·20	2·5	-1·72 (-4·40, 0·94)	27	0·69	0·0	4·67 (-19·26, 28·59)

[a] The number of individual association estimates.

[b] The fraction of between-study variance explained by study-level variables.

[c] Cross-sectional study vs. RCT / cohort study.

[d] Genital examination vs. self-report.

[e] ≤ 25 vs. ＞25 years old.

[f] ≤ 45% vs. ＞45%.

[g] Low risk bias vs. high risk bias.

Supplementary Table S4. Risk of bias assessment for cross-sectional studies – Newcastle-Ottawa Scale (adaptation)

Study (country)	Selection				Comparability based on design and analysis	Outcome		Total score	Overall risk of bias*
	Representative of the sample	Sample size	Non-respondents	Ascertainment of circumcision status		Assessment of outcome	Statistical test		
Bailey et al. 1999[12] (Uganda)	1	0	0	0	2	1	1	5	Low
Frisch et al. 2011[27] (Denmark)	1	0	0	0	0	1	0	2	High
Ayiga et al. 2011[13] (Botswana)	1	0	0	0	0	1	0	2	High
Forbes et al. 2012[14] (Tanzania)	1	0	0	0	0	1	1	3	High
Auvert et al. 2013[15] (South Africa)	1	0	0	1	0	1	1	4	High
Galbraith et al. 2014[16] (Kenya)	1	0	0	0	0	1	0	2	High
Balekang et al. 2016[17] (Botswana)	1	0	0	0	0	1	0	2	High
Kibira et al. 2016[18] (Uganda)	1	0	0	0	0	1	1	3	High
Zungu et al. 2016[19] (South Africa)	1	0	0	0	0	1	1	3	High
George et al. 2017[20] (South Africa)	1	0	1	0	0	1	0	3	High
Westercamp et al. 2017[21] (Kenya)	1	0	0	0	0	1	0	2	High
Ortblad et al. 2019[22] (South Africa)	1	0	1	0	0	1	1	4	High
Kufa et al. 2020[23] (South Africa)	1	0	1	1	0	2	1	6	Low
Wei et al. 2018[24] (China)	1	1	1	1	1	1	0	6	Low
Mwandi et al. 2012[25] (Kenya)	1	1	1	0	1	1	0	5	Low
Reed et al. 2012[26] (Swaziland)	1	1	1	0	1	1	0	5	Low

* High risk: score ≤ 4; low risk: score ≥ 5.

Supplementary Table S5. Risk of bias assessment for cohort studies – Newcastle-Ottawa Scale (adaptation)

Source	Selection				Comparability based on design and analysis	Outcome			Total score	Overall risk of bias
	Representative of the sample	Selection of the non-intervention cohort	Ascertainment of circumcision status	Demonstration that outcome of interest was not present at start of study		Assessment of outcome	Was follow up long enough for outcomes to occur	Adequacy of follow up of cohorts		
Kong et al. 2012[1] (Uganda)	1	1	1	1	2	1	1	0	8	Low
Feldblum et al. 2015[2] (Kenya)	0	0	1	1	1	1	1	1	6	Low
Mukudu et al. 2019[3] (South Africa)	0	0	1	1	2	1	1	1	7	Low
Agot et al. 2007[4] (Kenya)	1	1	1	1	1	1	1	1	8	Low
Westercamp et al. 2014[7] (Kenya)	1	1	1	1	0	1	1	0	6	Low
Kagaayi et al. 2016[8] (Uganda)	1	1	1	1	1	1	1	1	8	Low
Govender et al. 2018[9] (South Africa)	1	1	1	1	0	1	1	0	6	Low
Brito et al. 2017[10] (Dominican)	0	0	1	1	2	1	1	1	7	Low

High risk: score ≤ 4; low risk: score ≥ 5.

Supplementary Table S6. Risk of bias assessment for interventional studies - Cochrane Collaboration's tool

Study (country)	Random sequence generation	Allocation concealment	Blinding of outcome assessment	Incomplete outcome data	Selective reporting	Other forms of bias	Overall risk of bias
Tarnaud et al. 2011[11] (South Africa)	Low	Unclear	Low	Low	Low	Low [a]	Low
Bailey et al. 2007[5] (Kenya)	Low	Unclear	Unclear	Low	Low	Low [a]	Low
Gray et al. 2007[6] (Uganda)	Low	Unclear	Unclear	Low	Low	Low [a]	Low

[a] If a trial terminated early but substantial evidence had been collected, it is considered as low risk of bias.

Supplementary Table S7. Subgroup meta-analyses of studies conducted after 2007 of the association between MMC and HIV risk compensation among heterosexual men

	Condomless sex				Multiple sex partners			
	Reports (n)	Men (n)	Pooled OR (95%CI)	I-squared, (%)	Reports (n)	Men (n)	Pooled OR (95%CI)	I-squared, (%)
**Income of country**								
High	0	0	-	-	0	0	-	-
Low and Middle	24	46892	0·90 (0·76, 1·06)	90·5	21	52675	0·99 (0·84, 1·16)	89·7
**Study design**								
Cohort / RCT	7	7804	0·99 (0·75, 1·29)	79·5	7	9595	1·03 (0·87, 1·21)	56·9
Cross-sectional	17	39088	0·86(0·70, 1·06)	92·5	14	43080	0·96 (0·77, 1·20)	92·8
**Mean or median age, year**								
≤ 25	9	23053	0·82 (0·63, 1·08)	93·3	7	17721	1·03 (0·87, 1·22)	71·6
> 25	8	11891	0·94 (0·77, 1·15)	69·3	8	14069	0·96 (0·73, 1·26)	84·5
**Recruitment setting**								
Non-clinic-based	22	46077	0·88 (0·74, 1·04)	90·7	19	51860	0·96 (0·81, 1·14)	90·5
Clinic-based	2	815	1·22 (0·41, 3·62)	92·7	2	815	1·34 (0·77, 2·32)	70·5
**Circumcision assessment**								
Genital examination	13	17384	0·93 (0·71, 1·21)	91·9	12	21788	0·99 (0·87, 1·12)	63·9
Self-reported	10	29279	0·85 (0·69, 1·05)	89·0	8	30585	0·94 (0·65, 1·36)	95·5
**Circumcision, %**								
≤ 45	13	30280	0·88 (0·70, 1·11)	92·1	11	35972	1·01 (0·79, 1·30)	92·0
> 45	11	16612	0·92 (0·73, 1·16)	87·4	10	16703	0·96 (0·77, 1·18)	84·1
**Risk of bias**								
Low	11	21795	0·98 (0·80, 1·21)	81·8	10	15870	0·96 (0·77, 1·20)	81·8
High	13	25097	0·84 (0·65, 1·07)	93·5	11	36805	1·01 (0·80, 1·27)	92·3
**Married/cohabiting, %**								
≤ 50	6	15330	0·69 (0·45, 1·05)	96·0	6	18498	0·85 (0·65, 1·10)	89·7
> 50	8	16241	1·02 (0·84, 1·24)	70·1	8	31592	1·00 (0·72, 1·37)	92·2
**Priority countries for MMC**	21	38511	0·90 (0·75, 1·09)	91·6	19	51368	0·99 (0·83, 1·17)	90·7
**All samples**	**24**	**46892**	**0·90 (0·76, 1·06)**	**90·5**	**21**	**52675**	**0·99 (0·84, 1·16)**	**89·7**

Supplementary Figure S1. Sensitivity analysis of OR estimates on the association between MMC and condomless sex among heterosexual men

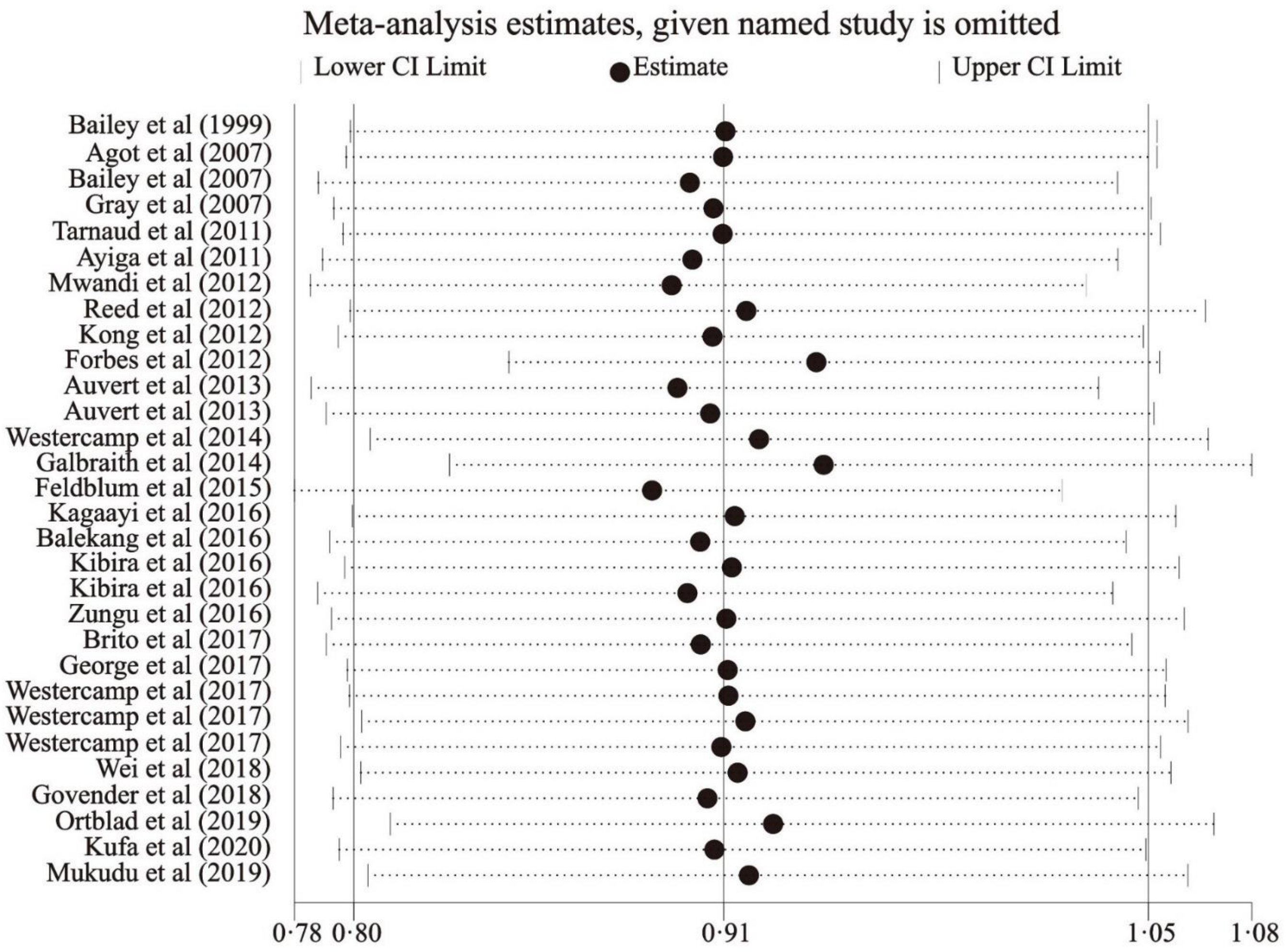

Supplementary Figure S2. Sensitivity analysis of OR estimates on the association between MMC and multiple sex partners among heterosexual men

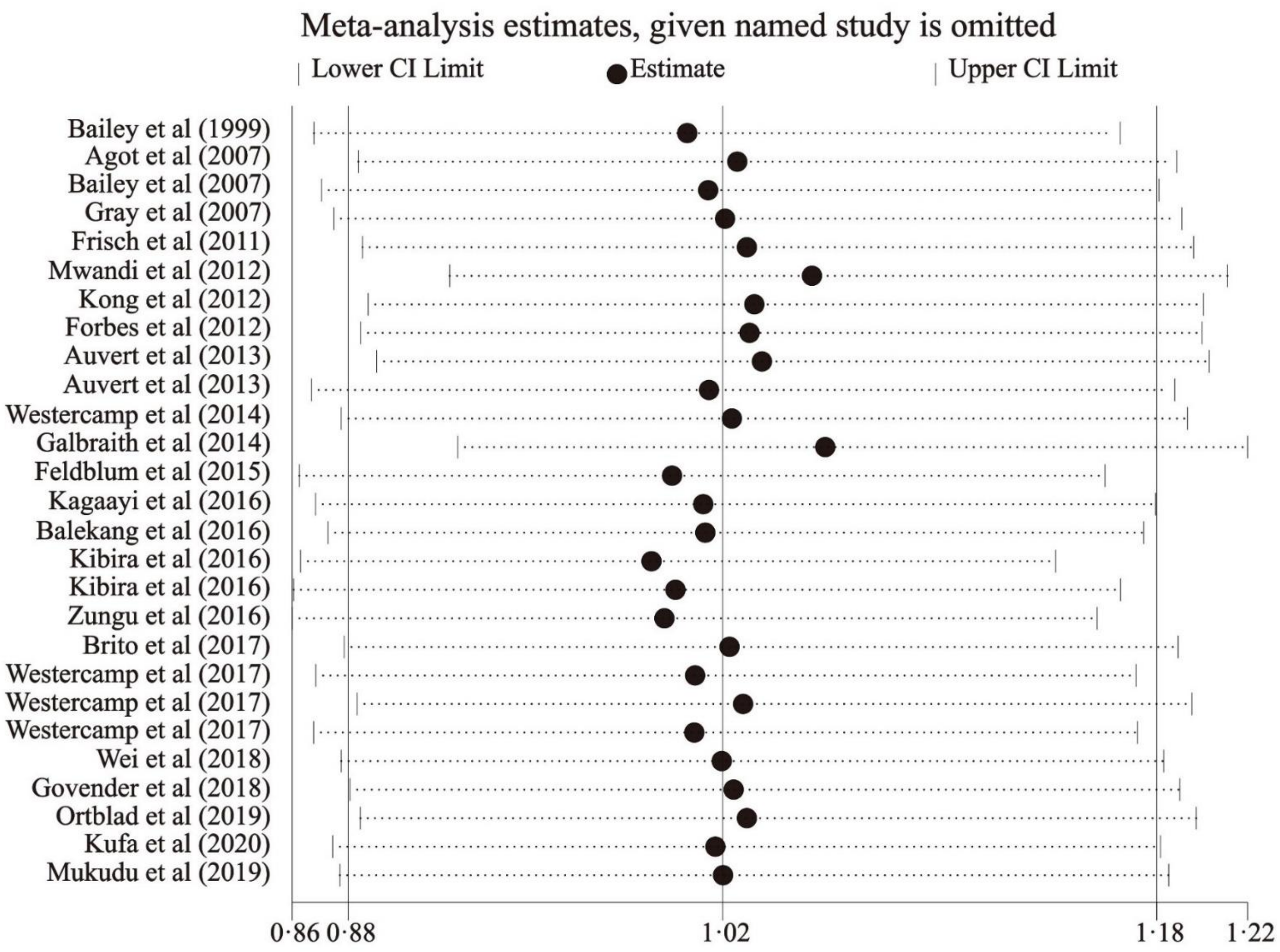

Supplementary Figure S3. Funnel plot of the log OR estimates of the association between MMC and condomless sex among heterosexual men

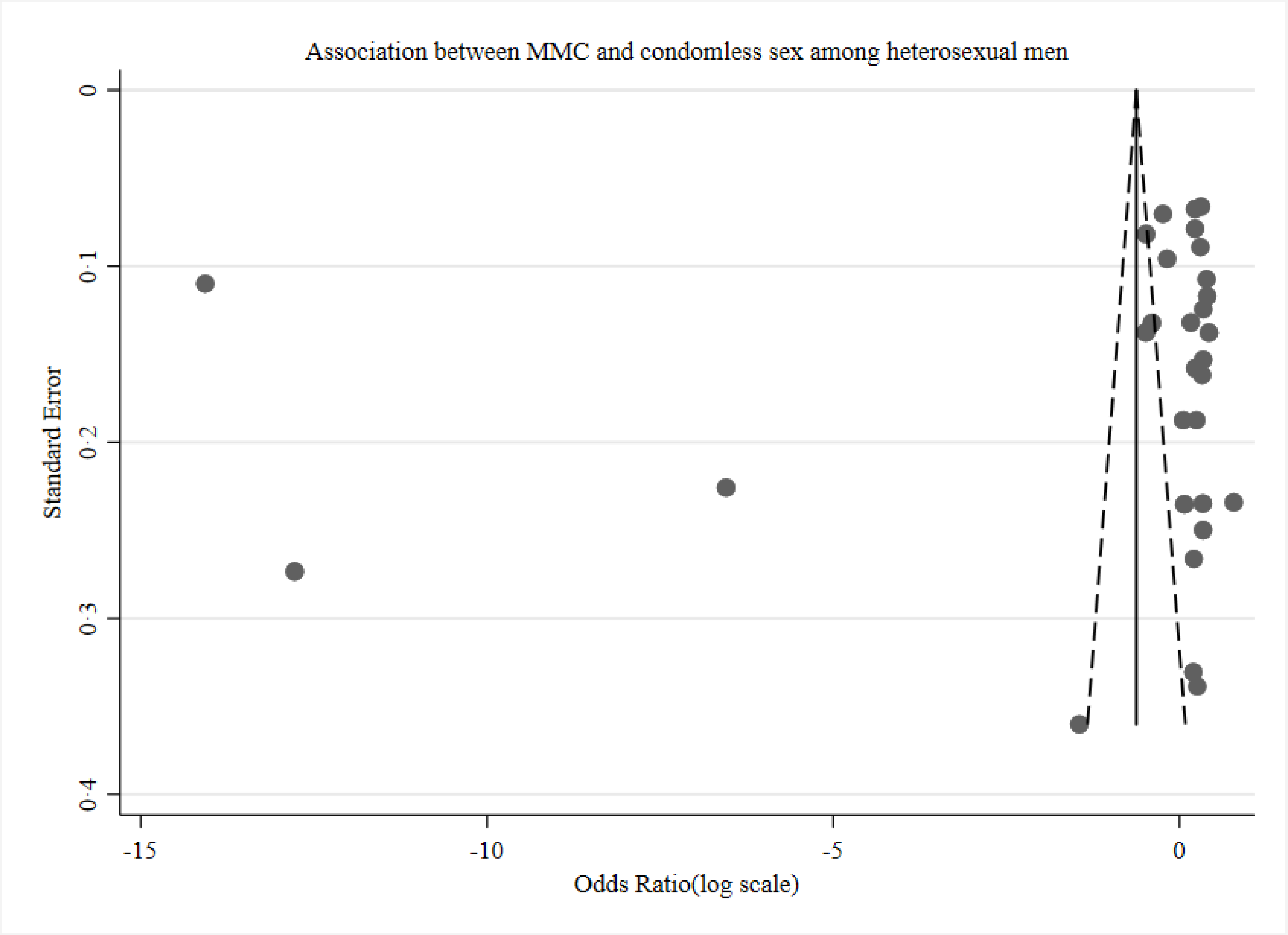

Supplementary Figure S4. Funnel plot of the log OR estimates of the association between MMC and multiple sex partners among heterosexual men

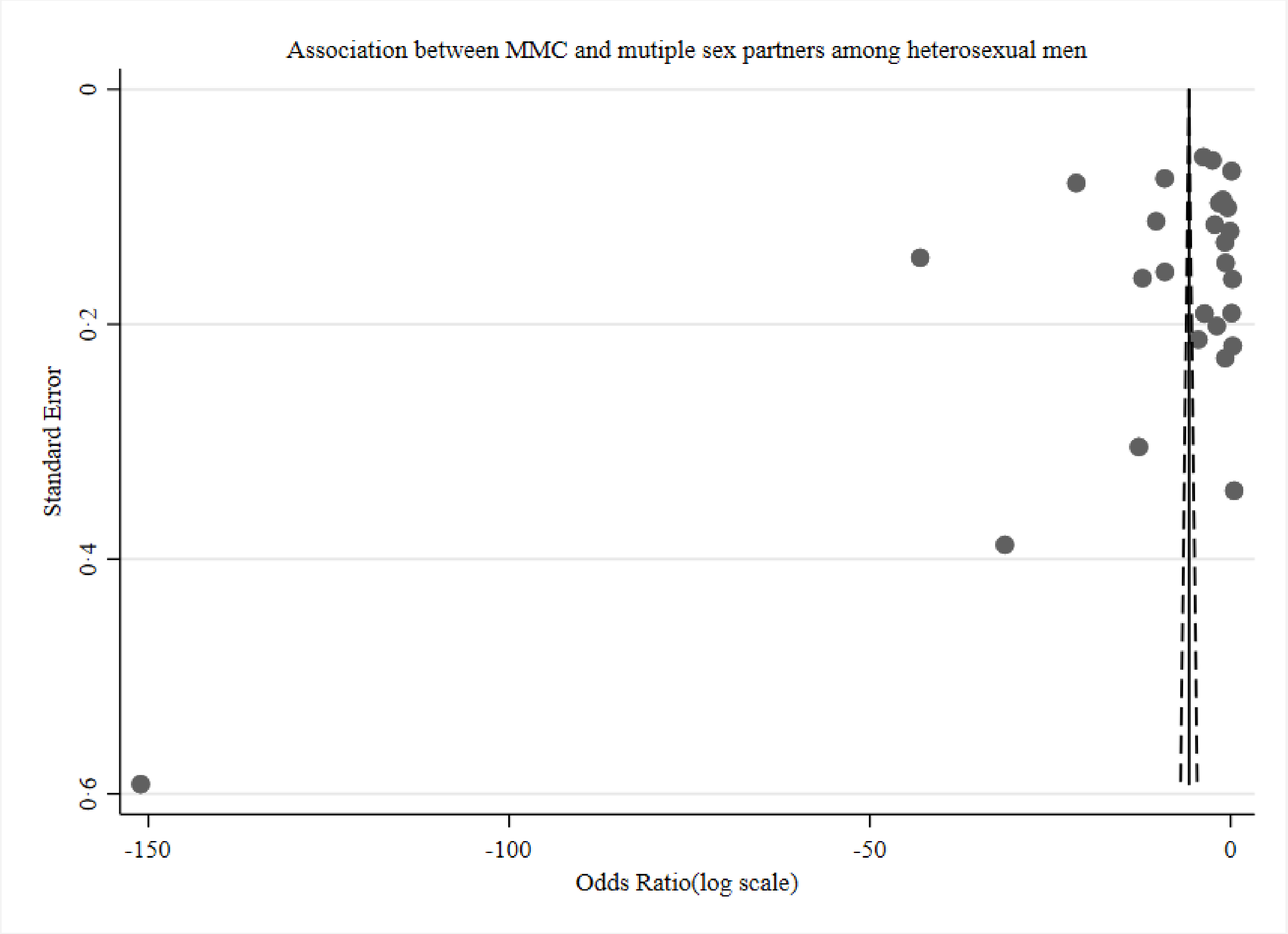

References:

1. Kong X, Kigozi G, Nalugoda F, et al. Assessment of changes in risk behaviors during 3 years of posttrial follow-up of male circumcision trial participants uncircumcised at trial closure in Rakai, Uganda. *Am J Epidemiol* 2012; **176**(10): 875-85.
2. Feldblum PJ, Okech J, Ochieng R, et al. Longer-Term Follow-Up of Kenyan Men Circumcised Using the ShangRing Device. *PloS one* 2015; **10**(9): e0137510.
3. Mukudu H, Dietrich J, Otwombe K, et al. Voluntary medical male circumcision (VMMC) for prevention of heterosexual transmission of HIV and risk compensation in adult males in Soweto: Findings from a programmatic setting. *PloS one* 2019; **14**(3): e0213571.
4. Agot KE, Kiarie JN, Nguyen HQ, Odhiambo JO, Onyango TM, Weiss NS. Male circumcision in Siaya and Bondo Districts, Kenya: prospective cohort study to assess behavioral disinhibition following circumcision. *J Acquir Immune Defic Syndr* 2007; **44**(1): 66-70.
5. Bailey RC, Moses S, Parker CB, et al. Male circumcision for HIV prevention in young men in Kisumu, Kenya: a randomised controlled trial. *Lancet* 2007; **369**(9562): 643-56.
6. Gray RH, Kigozi G, Serwadda D, et al. Male circumcision for HIV prevention in men in Rakai, Uganda: a randomised trial. *Lancet* 2007; **369**(9562): 657-66.
7. Westercamp N, Agot K, Jaoko W, Bailey RC. Risk compensation following male circumcision: results from a two-year prospective cohort study of recently circumcised and uncircumcised men in Nyanza Province, Kenya. *AIDS Behav* 2014; **18**(9): 1764-75.
8. Kagaayi J, Kong X, Kigozi G, et al. Self-selection of male circumcision clients and behaviors following circumcision in a service program in Uganda. *Aids* 2016; **30**(13): 2125-9.
9. Govender K, George G, Beckett S, Montague C, Frohlich J. Risk Compensation Following Medical Male Circumcision: Results from a 1-Year Prospective Cohort Study of Young School-Going Men in KwaZulu-Natal, South Africa. *Int J Behav Med* 2018; **25**(1): 123-30.
10. Brito MO, Khosla S, Pananookooln S, et al. Sexual Pleasure and Function, Coital Trauma, and Sex Behaviors After Voluntary Medical Male Circumcision Among Men in the Dominican Republic. *J Sex Med* 2017; **14**(4): 526-34.
11. Tarnaud C, Lissouba P, Cutler E, Puren A, Taljaard D, Auvert B. Association of low-risk human papillomavirus infection with male circumcision in young men: results from a longitudinal study conducted in Orange Farm (South Africa). *Infect Dis Obstet Gynecol* 2011; **2011**: 567408.
12. Bailey RC, Neema S, Othieno R. Sexual behaviors and other HIV risk factors in circumcised and uncircumcised men in Uganda. *J Acquir Immune Defic Syndr* 1999; **22**(3): 294-301.
13. Ayiga N, Letamo G. Impact of male circumcision on HIV risk compensation through the impediment of condom use in Botswana. *Afr Health Sci* 2011; **11**(4): 550-9.
14. Forbes HJ, Doyle AM, Maganja K, et al. Rapid increase in prevalence of male circumcision in rural Tanzania in the absence of a promotional campaign. *PloS one* 2012; **7**(7): e40507.
15. Auvert B, Taljaard D, Rech D, et al. Association of the ANRS-12126 male circumcision project with HIV levels among men in a South African township: evaluation of effectiveness using cross-sectional surveys. *PLoS Med* 2013; **10**(9): e1001509.
16. Galbraith JS, Ochieng A, Mwalili S, et al. Status of voluntary medical male circumcision in Kenya: findings from 2 nationally representative surveys in Kenya, 2007 and 2012. *J Acquir Immune Defic Syndr* 2014; **66 Suppl 1**: S37-45.
17. Balekang GB, Dintwa KF. A comparison of risky sexual behaviours between circumcised and uncircumcised men aged 30-44 years in Botswana. *Afr Health Sci* 2016; **16**(1): 105-15.
18. Kibira SP, Sandoy IF, Daniel M, Atuyambe LM, Makumbi FE. A comparison of sexual risk behaviours and HIV seroprevalence among circumcised and uncircumcised men before and

after implementation of the safe male circumcision programme in Uganda. *BMC public health* 2016; **16**: 7.

19. Zungu NP, Simbayi LC, Mabaso M, et al. HIV risk perception and behavior among medically and traditionally circumcised males in South Africa. *BMC public health* 2016; **16**: 357.

20. George G, Govender K, Beckett S, Montague C, Frohlich J. Factors associated with the take-up of voluntary medical male circumcision amongst learners in rural KwaZulu-Natal. *Afr J AIDS Res* 2017; **16**(3): 251-6.

21. Westercamp M, Jaoko W, Mehta S, Abuor P, Siambe P, Bailey RC. Changes in Male Circumcision Prevalence and Risk Compensation in the Kisumu, Kenya Population, 2008-2013. *J Acquir Immune Defic Syndr* 2017; **74**(2): e30-e7.

22. Ortblad KF, Harling G, Chimbindi N, Tanser F, Salomon JA, Bärnighausen T. Does Incident Circumcision Lead to Risk Compensation? Evidence from a Population Cohort in KwaZulu-Natal, South Africa. *J Acquir Immune Defic Syndr* 2019; **80**(3): 269-75.

23. Kufa T, Radebe F, Maseko V, Puren A, Kularatne R. Medical Male Circumcision and Associations Among Sexually Transmitted Infections Service Attendees. *AIDS Behav* 2020; **24**(5): 1422-31.

24. Wei FX, Guo M, Ma XJ, et al. The impact of male circumcision on the natural history of genital HPV infection: a prospective cohort study. *Chi J Prev Med* 2018; **52**(5): 486-92.

25. Mwandi Z, Bunnell R, Cherutich P, et al. Male circumcision programmes in Kenya: lessons from the Kenya AIDS Indicator Survey 2007. *B World Health Organ* 2012; **90**(9): 642-51.

26. Reed JB, Mirira M, Grund J, et al. Male circumcision in Swaziland: Demographics, behaviours and HIV prevalence. *J Int Aids Soc* 2012; **15**: 137.

27. Frisch M, Lindholm M, Gronbaek M. Male circumcision and sexual function in men and women: a survey-based, cross-sectional study in Denmark. *Int J Epidemiol* 2011; **40**(5): 1367-81.
